# Electronic structure, global reactivity descriptors and nonlinear optical properties of glycine interacted with ZnO, MgO and CaO for bacterial detection

**DOI:** 10.1038/s41598-024-72846-6

**Published:** 2024-10-01

**Authors:** Noha M. Sabry, Rania Badry, Fagr Kh. Abdel-Gawad, Hanan Elhaes, Medhat A. Ibrahim

**Affiliations:** 1 Water Pollution Research Department, Environment and Climate Change Research Institute, National Research Centre, 33 El-Bohouth St., Dokki, Giza, 12622 Egypt; 2 Center of Excellence for Research and Applied Studies on Climate Change and Sustainable Development, National Research Centre (NRC), 33 El Bohouth St. Dokki, Giza, 12622 Egypt; 3 Physics Department, Faculty of Women for Arts, Science and Education, Ain Shams University, 11757 Cairo, Egypt; 4 Spectroscopy Department, National Research Centre, 33 El-Bohouth St., 12622, Dokki, Giza, Egypt; 5 Molecular Modeling and Spectroscopy Laboratory, Centre of Excellence for Advanced Science, National Research Centre, 33 El-Bohouth St., 12622, Dokki, Giza, Egypt

**Keywords:** Glycine, Metal oxides, DFT, FTIR, UV-vis., Reactivity descriptors, NLO parameters, And antibacterial activity, Biophysics, Environmental sciences, Materials science

## Abstract

Modern laboratory medicine relies on analytical instruments for bacterial detection, focusing on biosensors and optical sensors for early disease diagnosis and treatment. Thus, Density Functional Theory (DFT) was utilized to study the reactivity of glycine interacted with metal oxides (ZnO, MgO, and CaO) for bacterial detection. Total dipole moment (TDM), frontier molecular orbitals (FMOs), FTIR spectroscopic data, electronic transition states, chemical reactivity descriptors, nonlinear optical (NLO) characteristics, and molecular electrostatic potential (MESP) were all investigated at the B3LYP/6–31G(d, p) level using DFT and Time-Dependent DFT (TD-DFT). The Coulomb-attenuating approach (CAM-B3LYP) was utilized to obtain theoretical electronic absorption spectra with the 6-31G(d, p) basis set to be more accurate than alternative quantum chemical calculation approaches, showing good agreement with the experimental data. The TDM and FMO investigation showed that glycine/CaO model has the highest TDM (10.129Debye) and lowest band gap (1.643 eV). The DFT computed IR and the experimental FTIR are consistent. The calculated UV-vis spectra showed a red shift with an increase in polarity following an increase in the absorption wavelength due to the interaction with ZnO, MgO, and CaO. Among the five solvents of water, methanol, ethanol, DMSO and acetone, the water and DMSO enhances the UV-Vis absorption. Glycine/CaO model showed high linear polarizability (14.629 × 10^−24^esu) and first hyperpolarizability (23.117 × 10^−30^esu), indicating its potential for nonlinear optical applications. The results showed that all model molecules, particularly glycine/CaO, contribute significantly to the development of materials with potential NLO features for sensor and optoelectronic applications. Additionally, MESP confirmed the increased electronegativity of the studied structures. Additionally, glycine/ZnO nanocomposite was synthesized and characterized using IR and UV-visible spectroscopy to determine their structural and spectroscopic features. It was discovered that there was good agreement between the DFT computed findings and the related experimental data. The antibacterial activity of glycine/ZnO nanocomposites against *Staphylococcus aureus* (*S. aureus)* and *Pseudomonas aeruginosa* were studied in terms of concentration and time. The results showed that increasing the concentration of glycine/ZnO nanocomposite significantly enhanced its antibacterial efficacy by lowering optical density. Notably, *Pseudomonas aeruginosa* exhibited lower susceptibility to the nanocomposite compared to *S. aureus*, requiring higher concentrations for effective bactericidal action. In summary, this study contributes novel insights into the dual functionality of glycine-metal oxide complexes, with significant implications as optical biosensor for microbial detection.

## Introduction

 Humanity is being forced to search for new pathogen detection techniques due to the increase of infectious diseases and bacterial pollution of food and water. Bacterial identification is also a top priority due to the ever-increasing hazards to both personal and territory security. Since infectious diseases can account for as much as 70% of cases, infections are a major contributor to early mortality^1,2^. Microorganism identification and quantification have become essential for drug discovery, biodefense, and food safety research. For the protection of the public’s health and the environment, pathogens and indicator microorganisms must be found in water and food samples^3^.

Accurately identifying and detecting bacteria in food and water is crucial for prompt and effective treatment, as well as for the general public’s safety and health. In the long run, early detection of bacterial infections may lower sickness rates and stop the spread of potentially fatal epidemics. Conventional laboratory diagnosis techniques are labor-intensive, time-consuming, and expensive; they also call for specialized personnel and equipment. Significant advancements have been made in the last few years in the development of low-cost, quick, precise, and accurate techniques for the detection of bacteria^4–6^.

Biosensor systems, integrating advances in biomedicine, optoelectronics, microelectronics, and nanotechnology, provide a promising alternative to traditional bacterial detection methods^7^. Optical biosensors are particularly effective, as they measure physical changes like light absorption, reflection, and luminescence rather than relying on chemical reactions^8^. These sensors detect alterations in optical properties caused by the presence of bacteria or viruses, such as absorbance, refractive index, turbidity, and color. The development of biosensors, especially with the incorporation of various nanomaterials, has significantly enhanced their performance as efficient sensing platforms^9,10^.

It is generally known that nanomaterials such as ZnO, MgO, and CaO nanoparticles can be used to create the best sensors possible for a variety of hazardous contaminants ^11^. According to Khan et al. (2019), nanomaterials with a size range of 1–100 nm have special chemical and physical properties like optical, magnetic, electrical, and chemical flexibility^12^. For the past 20 years, these properties have made them an effective sensing platform in the field of analytical chemistry.

According to biological principles, ZnO nanostructured materials have been used in the cosmetic and sunscreen industries due to its appealing optical qualities and capacity to reflect, scatter, and absorb UV rays. ZnO nanoparticles have been used as food supplements^13–15^. ZnO nanostructured materials are ideal for biological applications such as antibacterial agents, medication delivery, bio-imaging probes, cancer treatment, and infectious disease treatment^16^.

Magnesium oxide (MgO) is an alkaline earth metal oxide. The industrial uses of MgO nanoparticles as catalysts, adsorbents, and catalytic supports are well recognized^17,18^. The MgO nanostructures function as extremely absorbent materials for enzyme immobilization in the majority of these biosensors. This makes MgO-based biosensors extremely sensitive possible^19^.

The advancement of organic materials, particularly amino acids (AAs), has revolutionized sensing technologies^20,21^. AAs, crucial macromolecules in all living organisms, play key roles in various biological functions and microbial resistance. They form proteins through molecular crystals held together by hydrogen bonds and salt bridges, influencing their mechanical, electrical, and optical properties^22,23^. AAs are valued for their biocompatibility, biodegradability, and electro-optical properties. Their ability to self-assemble into stable nanostructures like fibers, capsules, and nanotubes opens up new possibilities in materials and pharmaceutical sciences. Understanding the optical and electrical characteristics of AAs can drive innovation in nanostructure development^24,25^.

In recent years, various applications have been developed that combine amino acids and nanotechnology. For instance, functionalizing graphene with amino acids has created soluble nanostructures that can be utilized as ultrasensitive biosensors while preserving the conductivity of original graphene^26^. Stable fluorescent amino acid nanotubes constructed of tryptophan and tyrosine were produced, and chiral polymer nanoparticles using amino acids were used as nucleating agents for the selective crystallization of racemic amino acid mixtures^27^. Blue fluorescence was observed in carbon dots functionalized with glycine and L-valine; these dots were suggested as bioimaging probes^28^.

Glycine, the simplest amino acid with a lateral chain made up of a single hydrogen atom, is found in modest amounts in most proteins and functions as an inhibitory neurotransmitter in the central nervous system^29^. Glycine has been shown to be highly helpful in promoting both human and animal health as well as growth and wellbeing. Rather, elevated glycine concentrations cause lysis or profound morphological changes in a large number of bacteria. There isn’t study on how glycine can sense the bacterial pollution yet^30,31^.

The identification of novel biosensor materials and the determination of the electrical properties and vibrational frequencies of molecular systems depend heavily on computational investigations^32,33^. Among the theories that are now most helpful and flexible in this field of study is the density functional theory (DFT). It is now easier to explain the reactivity of both simple and complicated systems without going into great detail about the reaction pathway because to DFT, which makes it easy to comprehend chemical selectivity by looking at features that are isolated from a compound^34^. Global softness (S), global hardness (η), and electrophilicity (ω) are among the notions and descriptors of chemical reactivity that have been derived in this context using DFT^35,36^.

Infection by bacteria is one of the main issues with human health. Early detection of bacterial pathogens and efficient elimination of pathogenic germs are critical for preventing the spread of diseases caused by bacteria^37^. Accordingly, in this work, we created a novel nanocomposite material that has the ability to efficiently sense bacteria and treating pathogenic microorganisms. Accordingly, the two primary goals of this study are:1. Applying DFT to investigate electronic, IR, optical, biological descriptors, and NLO properties 2. Synthesis of glycine/metal oxide nanocomposites for the assessment of the antibacterial activity of glycine/ZnO nanocomposites against both two types of bacteria: *Staphylococcus aureus* (Gram-positive) and *Pseudomonas aeruginosa* (Gram-negative) using the UV-vis spectrophotometer. Understanding the electrical structure, biological activity, and NLO characteristics of glycine/metal oxide nanocomposites is made possible by this kind of research. The experimentally synthesized nanocomposites were subjected to theoretical calculations using the DFT and time-dependent density functional theory (TD-DFT). These calculations were frequently carried out by comparing the theoretically calculated spectroscopic results with the experimental spectroscopic data (FTIR and UV-vis absorption spectra) with in order to further validate the structure of the proposed models. Additionally, DFT calculations for different solvent characteristics have been discussed. For the purpose of assessing the NLO properties of glycine nanocomposites, the current study is essential. This work should pave the way for the creation of completely new glycine -based metal oxides with remarkable NLO characteristics.

## Experimental section

### Materials and instrumentation

The following list of reagents was used without any additional purification. The absolute ethanol (analytical reagent, 99.9%) and sodium hydroxide pellets (NaOH) were supplied by El Nasr Pharmaceutical Chemicals Co., Cairo, Egypt. Sigma-Aldrich in Germany provided the laboratory reagent, zinc acetate and glycine. The glassware was thoroughly cleaned with a soap solution and then thoroughly cleaned with deionized water.

The Fourier-transform infrared (FTIR) spectrophotometer was used to characterize the presence of ZnO nanoparticles in the nanocomposites. FTIR-ATR spectrophotometer (Bruker Vertex 70) was used to record the FTIR spectra, with 35 scans from 4000 to 400 cm^−1^ at a resolution of 4 cm^−1^. The diamond ATR attachment has a penetration depth of two meters when used in conjunction with a type II alpha diamond crystal. At a resolution of 4 cm^−1^, the same parameters were used to test the air versus the background. The samples were utilized without first being prepared. The sample was finely ground, combined with potassium bromide (KBr) using a mortar and pestle, and then formed into pellets prior to the measurement. At room temperature, the optical characteristics were examined using an ultraviolet/visible spectrophotometer (UV/Vis., V-570 UV/VIS/NIR, JASCO, Japan) in the wavelength range of 200–800 nm.

### ZnO Preparation

ZnO nanoparticles were prepared by the precipitation method as prepared in our previous work^13^, which involved dissolving 0.2 mol of zinc acetate in 100 ml of deionized water and vigorously stirring with a magnetic stirrer until it was completely dissolved. The zinc acetate solution was then heated at 60^o^C for about 30 min. 0.4 mol of NaOH was dissolved in 50 ml of deionized water (DW). The NaOH solution was added drop by drop to the previous solution of zinc acetate under strong stirring for three hours, resulting in white precipitation. The resulting suspension was then repeatedly washed with deionized water and filtered. The sample was then dried further by being placed in a dryer at 80^o^C for 2:30 h. Lastly, the white precipitate was calcined for two hours at 500^o^C, which guarantees that there are no hydroxyl or carbonyl groups in the sample.

### Glycine/ZnO nanocomposite preparation

The glycine and glycine doped with 8wt% of ZnO NPs were prepared using the solution casting method. One gram of glycine (weighted using a digital mass balance) was dissolved in one hundred milliliters of deionized water. The glycine solution was agitated for two hours using a magnetic stirrer and bar until the glycine powder was completely dissolved. Subsequently, 4 wt% of ZnO NPs were added to the glycine solution separately and continuously agitated for two hours. To prevent ZnO from aggregating, glycine nanocomposite solution was placed in an ultrasonic water path for a duration of one hour. The reaction accuracy is validated by FTIR and UV-vis. analysis.

### Evaluation of antibacterial behavior of glycine/ZnO nanocomposites

The antibacterial activity of glycine and glycine/ZnO nanocomposites was evaluated against both Gram-positive and Gram-negative bacteria. The Gram-positive strains tested *Staphylococcus aureus* (ATCC 25923). For the Gram-negative strains, *Pseudomonas aeruginosa* (ATCC 10145) was used. To prepare stock cultures, the bacterial strains were inoculated onto Mueller Hinton Broth (MHB) (Himedia, India) and incubated at 37 °C for 24 h. Bacterial cultures were incubated with glycine alone and glycine/ZnO nanocomposites at concentrations of 50, 100, 300, and 500 µg/mL. MHB was used as a negative control, while 100 µg/mL kanamycin served as the positive control^38^.

### Calculation details

Molecular modeling computations were performed using Gaussian 09 computer package^39^. The input files were organized with Gauss View 5.0^40^ using the density functional theory (DFT). Gauss Sum program was utilized to interpret the data^41^. Glycine was completely optimized utilizing the DFT: B3LYP level of theory and the 6-31G(d, p) basis set combination in the gas phase, with no symmetry constraints. The Lee-Yang-Parr correlation functional (LYP) and Beck’s three-parameter hybrid exchange functional (B3) are combined to form the B3LYP functional. The basis set 6-31G (d, p) with “d” polarization functions on heavy atoms and “p” polarization functions on hydrogen atoms was used to more accurately describe polar bonding in molecules^42–44^.

Total dipole moment (TDM), frontier molecular orbitals (FMO), and solvent effect were conducted at the B3LYP/6-31G(d, p) level. A frequency study using the same theoretical level demonstrated the stability of optimized geometries. The absence of negative Eigen values across all estimated frequencies emphasizes the optimized geometries at real positive and true minimums in the potential energy surface^45^. The CAM-B3LYP Coulomb-attenuated functional was utilized to determine electronic excitation energies. This functional offers superior overall performance; there is no discernible correlation between excitation energy errors and the well-rounded, high-quality description of all excitation energy categories^46^. Time dependent- density functional theory (TD-DFT) at the CAM/B3LYP level of theory were used to estimate glycine/metal oxide nanocomposite’s photophysical parameters, which were combined with the 6-31G(d, p) basis set. At the B3LYP/6-31G(d, p) theoretical level, global reactivity descriptors, nonlinear optical parameters (NLO) and molecular electrostatic potential (MESP) maps were conducted. The equations (from Eq. 1 to 10) used to calculate the global reactivity descriptors, meanwhile the NLO parameters are calculated using the equations from 11 to 13^35,36^.

1 $$\rm IE=-E_{HOMO}$$

2 $$\rm EA=-E_{LUMO}$$

3 $$\triangle \rm E=E_{HOMO}-E_{LUMO}$$

4 $$\rm \eta=Energy\, gap/2$$

5 $$\rm \mu=(E_{HOMO}+ E_{LUMO})/2$$

6 $$\rm S=1/ \eta$$

7 $$\rm \omega= \mu^2/2\eta$$

8 $$\rm N=1/\omega$$

9 $$\rm \chi=-\mu$$

10 $$\rm \triangle N_{max}=-\mu/\eta$$

11 $$\rm (\mu)=(\mu^2_x+\mu^2_y+\mu^2_z)^{1/2}$$

12 $$\rm (\langle\alpha\rangle)=1/3(\alpha_{xx}+\alpha_{yy}+\alpha_{zz}$$

13 $$\rm (\triangle\alpha)=((\alpha_{xx}-\alpha_{yy})^{2}+(\alpha_{yy}-\alpha_{zz})^{2}+(\alpha_{zz}-\alpha_{xx})^2)^{1/2}$$

$$\rm (\langle\beta\rangle=[\beta^2_x+\beta^2_y+\beta^2_z/2]^{1/2}$$

Where;

$$\rm \beta_x=\beta_{xxx}+\beta_{xyy}+\beta_{xzz},$$

$$\rm \beta_y=\beta_{yyy}+\beta_{xxy}+\beta_{yzz},$$

$$\rm \beta_z=\beta_{zzz}+\beta_{xxz}+\beta_{yyz},$$

Note that the conversion factors used to calculate α, β, HOMO, and LUMO energies in atomic and CGS units are as follows: 1 atomic unit (a.u.) = 0.1482 × 10^−24^ electrostatic unit (esu) for $\rm \langle\alpha\rangle$; 1 a.u. = 8.6393 × 10^−33^ esu for $\rm \langle\beta\rangle$; 1 a.u. = 27.2116 eV for HOMO and LUMO energies.

## Result and discussion

### Building model molecules

**Fig. 1:**
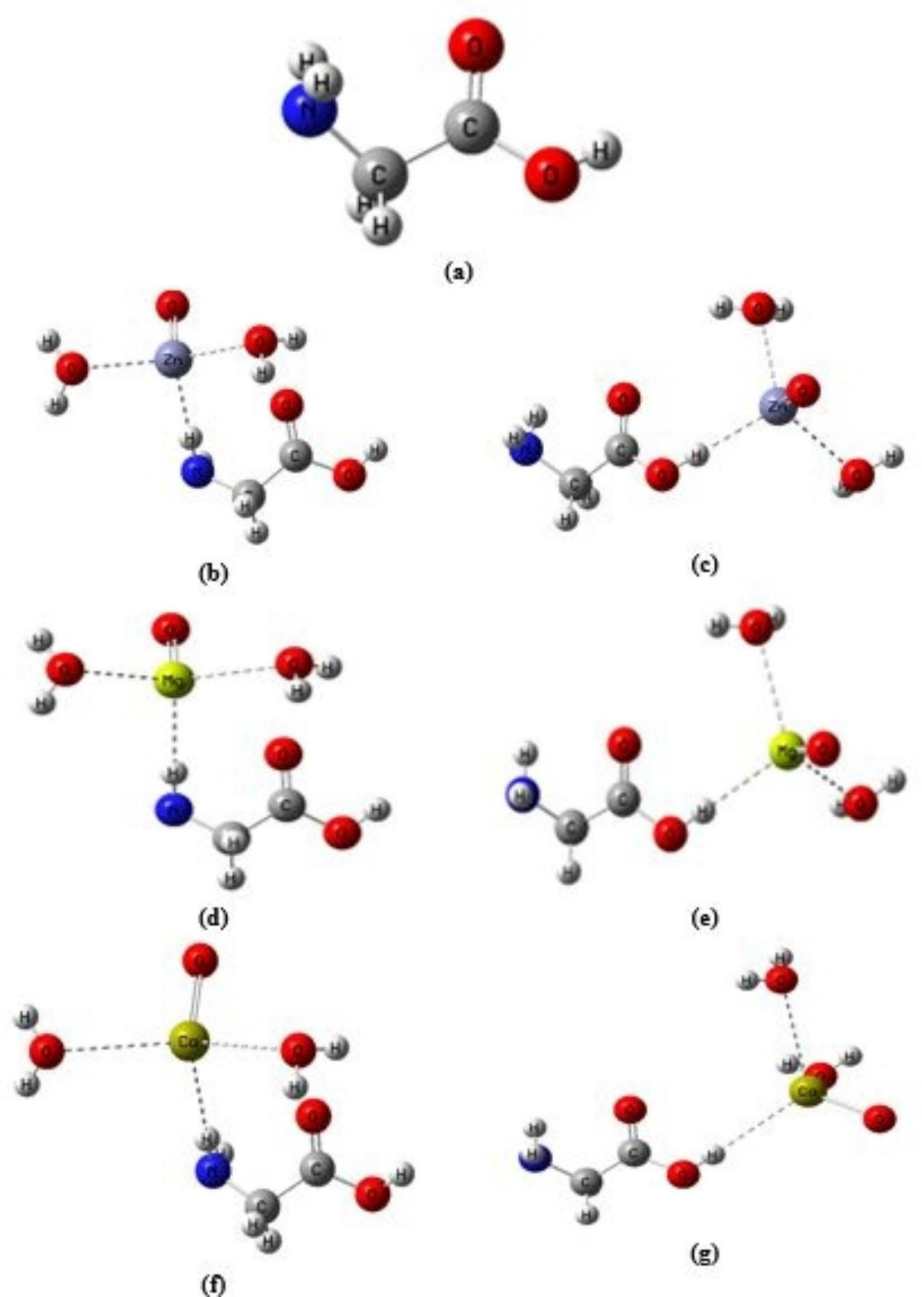
Optimized structure of **a**) glycine and glycine interacted with hydrated ZnO, MgO, and CaO through: **b**), **d**), and **f**) NH_2_ functional group and **c**), **e**), and **g**) COOH functional group.

One important aspect in molecular modeling is to describe the building of the studied model molecules. Figure 1-a illustrates the three active sites (NH_2_, OH, and = O) of the well-known amino acid glycine. One metal oxide molecule and one glycine molecule are intended to interact. The most active sides of contact, NH_2_ and OH, are probably how metal oxides will interact with glycine. Additionally, as illustrated in Fig. 1-b, c, d, e, f, and g, nanomaterials including ZnO, MgO, and CaO were selected to investigate their impacts on glycine characteristics and reactivity in the presence of two water molecules.

### Total dipole moment (TDM) for glycine/ metal oxides models

**Fig. 2:**
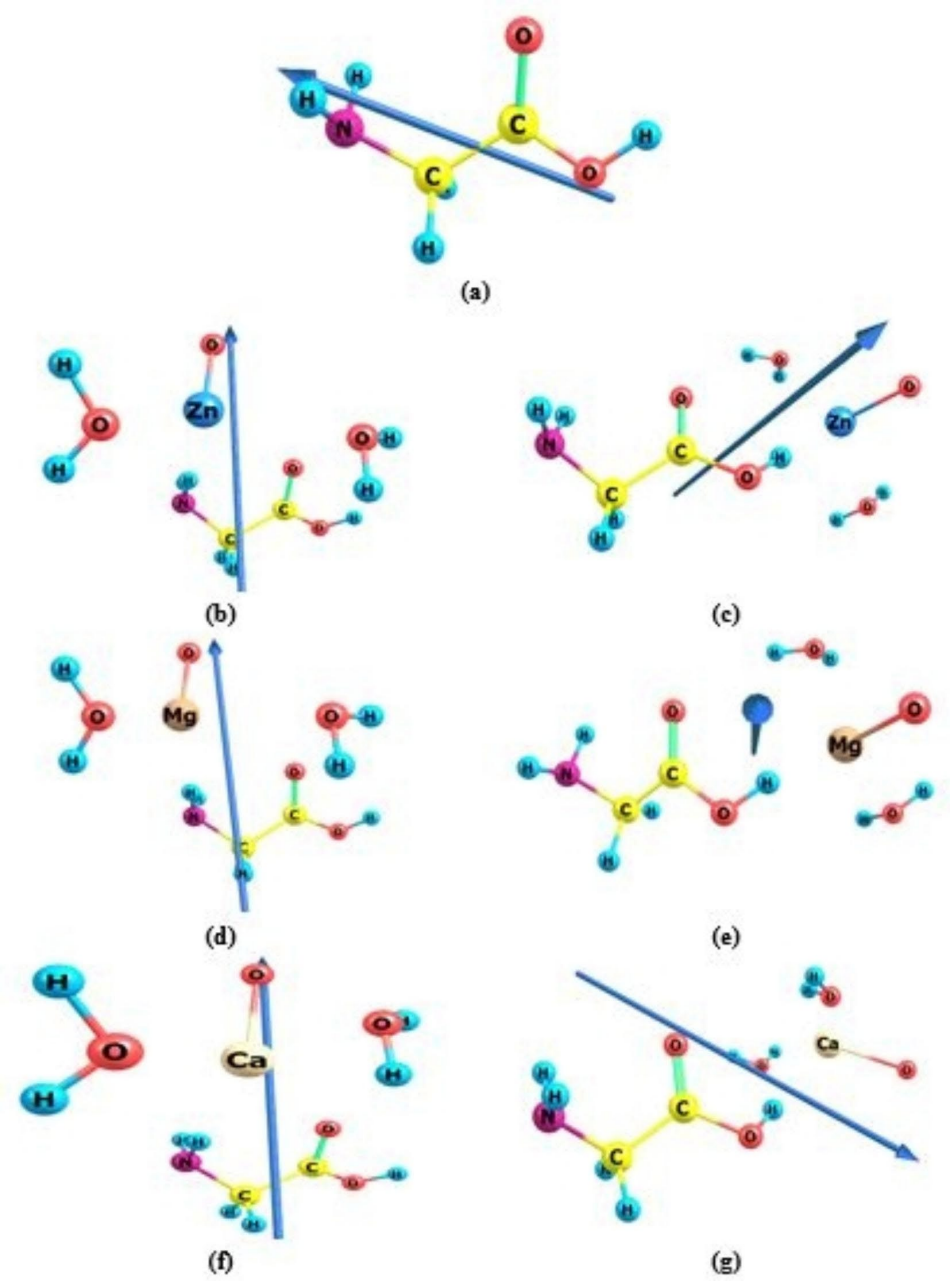
B3LYP/6-31G(d, p) calculated TDM vector of **a**) glycine and glycine interacted with hydrated ZnO, MgO, and CaO through: **b**), **d**), and **f**) NH_2_ functional group and **c**), **e**), and **g**) COOH functional group.

Table 1 displays the TDM results for the nanocomposite systems of glycine, glycine/ZnO, glycine/MgO, and glycine/CaO nanocomposite models. Interaction with the suggested metal oxides caused TDM to rise, suggesting that functionalized glycine is more polarizable than pure glycine. Figure 2 displays the vector of the dipole moment of glycine and glycine interacted with ZnO, MgO, and CaO through the two interaction mechanisms that were computed using the B3LYP/6-31G(d, p). Glycine interacted with ZnO throughout the NH_2_ and COOH functional groups, causing TDM to increase from 1.310 debye to 7.4457and 9.6404 Debye, respectively. In the meantime, TDM increased to 9.037 and 10.802 Debye when glycine interacted with MgO, indicating that the interaction went through the NH_2_ and COOH functional groups, respectively. Lastly, TDM increased to 9.144 and 10.1292Debye for the same sequence when glycine interacted with CaO. The elevated TDM values of glycine resulting from its interaction with the examined metal oxides validate the heightened reactivity of glycine. Moreover, the differences in TDM values of glycine resulting from its interactions with ZnO, MgO, and CaO validate the formation of hydrogen bonds between the hydrogen atom of glycine and the oxygen atom of the investigated metal oxides. Additionally, the outcomes verified that the most likely interaction between glycine and ZnO, MgO, and CaO is one that occurs via the COOH functional group.

**Table 1:** B3LYP/6-31G(d, p) calculated total dipole moment (TDM) as Debye and HOMO energy, LUMO energy, and HOMO/LUMO bandgap energy ($\:\varDelta\:$E) as eV for glycine/metal oxides including ZnO, MgO, and CaO with two water molecules in the gas phase and solvent.

Phase	Structure	TDM	E_HOMO_ (eV)	E_LUMO_ (eV)	$\:\varDelta\:$E (eV)
Gas	Glycine	1.310	-6.712	-0.583	6.129
	Glycine (NH_2_)-ZnO-H_2_O	7.446	-5.044	-2.569	2.476
	Glycine (COOH)-ZnO-H_2_O	9.640	-5.527	-3.108	2.418
	Glycine (NH_2_)-MgO-H_2_O	9.037	-4.366	-2.391	1.976
	Glycine (COOH)-MgO-H_2_O	10.802	-4.831	-2.904	1.926
	Glycine (NH_2_)-CaO-H_2_O	9.144	-3.734	-2.083	1.651
	Glycine (COOH)- CaO-H_2_O	10.129	-4.057	-2.414	1.643
Water	Glycine	1.612	-6.785	-0.574	6.211
	Glycine (NH_2_)-ZnO-H_2_O	13.973	-4.530	-1.154	3.376
	Glycine (COOH)-ZnO-H_2_O	15.507	-4.646	-1.426	3.219
	Glycine (NH_2_)-MgO-H_2_O	11.494	-5.201	-1.547	3.654
	Glycine (COOH)-MgO-H_2_O	15.507	-4.646	-1.426	3.219
	Glycine (NH_2_)-CaO-H_2_O	16.869	-3.214	-0.848	2.366
	Glycine (COOH)- CaO-H_2_O	17.107	-3.249	-0.935	2.314
Methanol	Glycine	1.603	-6.782	-0.575	6.208
	Glycine (NH_2_)-ZnO-H_2_O	13.814	-4.511	-1.208	3.303
	Glycine (COOH)-ZnO-H_2_O	15.359	-4.642	-1.492	3.150
	Glycine (NH_2_)-MgO-H_2_O	11.341	-5.196	-1.606	3.590
	Glycine (COOH)-MgO-H_2_O	12.833	-5.384	-1.920	3.465
	Glycine (NH_2_)-CaO-H_2_O	16.661	-3.210	-0.888	2.322
	Glycine (COOH)- CaO-H_2_O	16.914	-3.255	-0.986	2.269
Ethanol	Glycine	1.598	-6.780	-0.575	6.205
	Glycine (NH_2_)-ZnO-H_2_O	13.730	-4.501	-1.236	3.265
	Glycine (COOH)-ZnO-H_2_O	15.281	-4.641	-1.527	3.114
	Glycine (NH_2_)-MgO-H_2_O	11.262	-5.194	-1.636	3.557
	Glycine (COOH)-MgO-H_2_O	12.762	-5.389	-1.954	3.435
	Glycine (NH_2_)-CaO-H_2_O	16.549	-3.210	-0.910	2.299
	Glycine (COOH)- CaO-H_2_O	16.810	-3.259	-1.013	2.246
DMSO	Glycine	1.608	-6.783	-0.574	6.209
	Glycine (NH_2_)-ZnO-H_2_O	13.896	-4.520	-1.180	3.340
	Glycine (COOH)-ZnO-H_2_O	15.436	-4.644	-1.458	3.185
	Glycine (NH_2_)-MgO-H_2_O	11.419	-5.199	-1.576	3.622
	Glycine (COOH)-MgO-H_2_O	12.903	-5.380	-1.886	3.494
	Glycine (NH_2_)-CaO-H_2_O	16.769	-3.212	-0.867	2.344
	Glycine (COOH)- CaO-H_2_O	17.014	-3.252	-0.959	2.292
Acetone	Glycine	1.594	-6.779	-0.575	6.204
	Glycine (NH_2_)-ZnO-H_2_O	13.657	-4.493	-1.261	3.232
	Glycine (COOH)-ZnO-H_2_O	15.212	-4.640	-1.557	3.083
	Glycine (NH_2_)-MgO-H_2_O	11.193	-5.191	-1.662	3.529
	Glycine (COOH)-MgO-H_2_O	12.700	-5.393	-1.983	3.410
	Glycine (NH_2_)-CaO-H_2_O	16.450	-3.208	-0.929	2.279
	Glycine (COOH)- CaO-H_2_O	16.718	-3.263	-1.037	2.226

Additionally, the effect of solvent on the band gap energy of glycine and its nanocomposites is studied at the B3LYP/6-31G(d, p) level of theory. When a substance’s energy gap is smaller, it can interact with biological systems more effectively due to stronger intramolecular electron transfer (boosted bioactivity)^47^. The variation of TDM, HOMO, LUMO, and HOMO-LUMO energy for gas phase and various solvents is shown in Table 1. In contrast to the liquid phase, the gas phase is where HOMO is stable, and the LUMO region is where destabilization takes place. The reactivity hierarchy of the glycine/metal oxide nanocomposite in different solvent phases has been determined by preceding factors. For glycine model, water has the largest band gap value (6.211 eV) when compared to other solvents. Similarly, for glycine interacted with ZnO, MgO, and CaO through the two interaction mechanisms water has the largest band gap value. It is possible that in solvents with increasing polarity, molecule stability rises with improved van der Waals contacts. Protic solvents, such as water, also aid in stabilizing the molecule by forming hydrogen bonds. Water, the solvent with the highest polarity, makes glycine more stable and resistant to side effects by increasing its chemical hardness and decreasing its chemical softness.

### Frontier molecular orbital (FMO) analysis

**Fig. 3:**
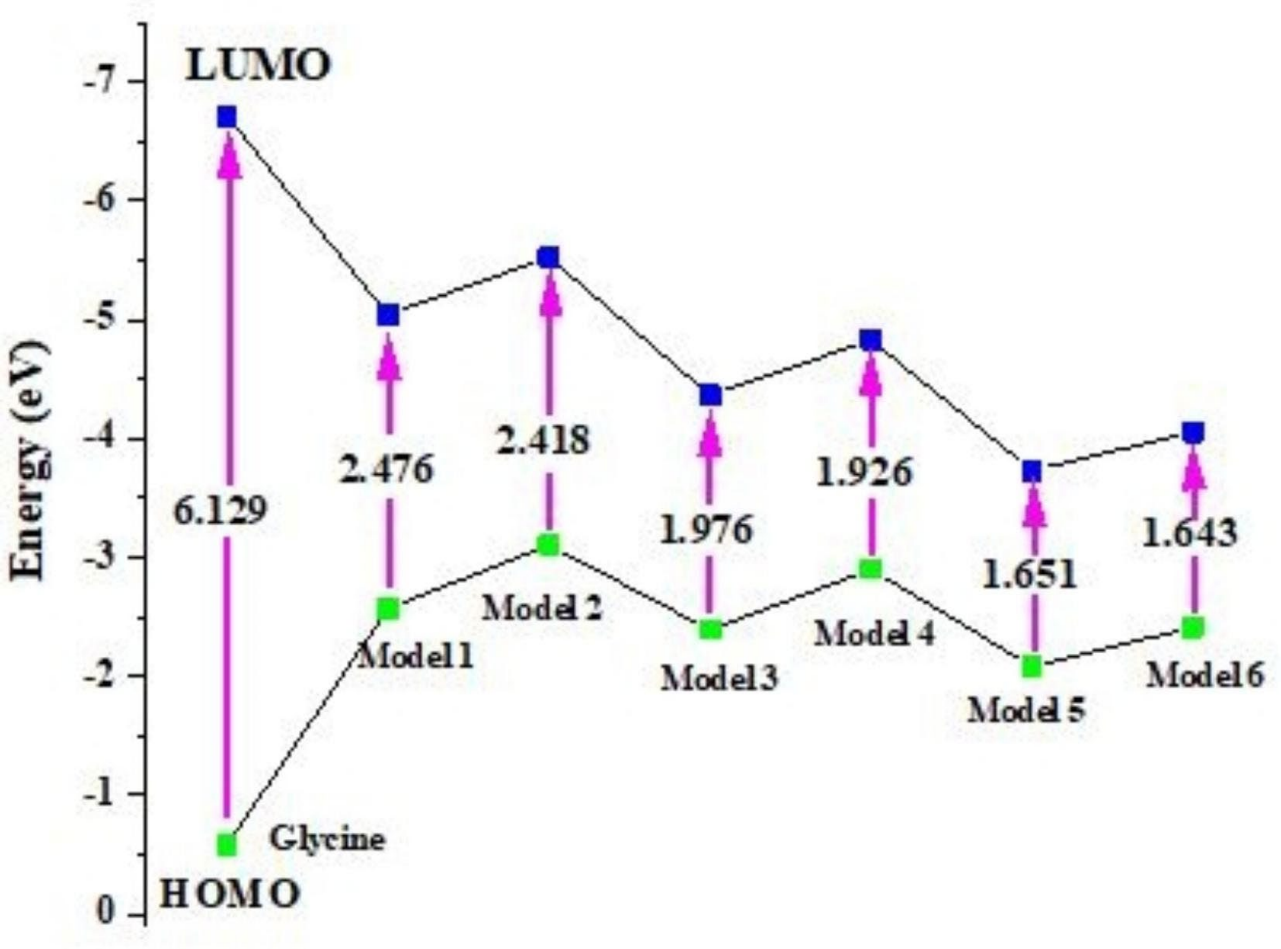
Graphical representation of HOMO, LUMO and energy gap of Glycine, Glycine (NH_2_)-ZnO-H_2_O (Model 1), Glycine (COOH)-ZnO-H_2_O (Model 2), Glycine (NH_2_)-MgO-H_2_O (Model 3), Glycine (COOH)-MgO-H_2_O (Model 4), Glycine (NH_2_)-CaO-H_2_O (Model 5), and Glycine (COOH)- CaO-H_2_O (Model 6) at B3LYP/6-31G(d, p) level of theory.

 FMO analysis is a powerful method for assessing the optical and electrical properties of compounds, including the likelihood of intramolecular charge transfer (ICT)^48,49^. The valence band is represented by HOMO, which can donate electrons, and the conduction band by LUMO, which can accept electrons. FMOs are a significant source of transition energy when an electron excites from the HOMO to the LUMO. Models’ energy differential (ΔE = E_LUMO_-E_HOMO_) affects their stability and reactivity. Reduced energy difference (ΔE) values increase molecule polarizability, leading to improve nonlinear optical (NLO) response^50^. Figure 3 showes the graphical representation of the HOMO-LUMO bandgap energy variation of glycine, glycine/ZnO, glycine/MgO, and glycine/CaO model molecules. The E_LUMO_, E_HOMO_, and ΔE values for glycine and glycine interacted with ZnO, MgO, and CaO were calculated, and the results are tabulated in Table 1.

Table 1 shows that various functional groups in glycine structure significantly affect the band gap of produced molecules. Model molecules with high ΔE require more energy to shift from HOMO to LUMO, making them not suitable for nonlinear optical (NLO) devices^51,52^.

Molecules having lower band gaps are advantageous in sensor and opto-electronic applications. In these model molecules, HOMO and LUMO energies are changed, resulting in varying ΔE values. The HOMO energies of glycine and glycine interacted with ZnO, MgO, and CaO throughout the NH_2_ functional group are − 5.044, -4.366, and − 3.734 eV, respectively, while the LUMO energies are − 2.569, -2.391, and − 2.083 eV, respectively. However, for the interaction of glycine ZnO, MgO, and CaO with proceeds through the COOH functional group, the HOMO energies are 2.418, -4.831, and − 4.057 eV, respectively, while the LUMO energies are − 3.108, -2.904, and − 2.414 eV, respectively. The HOMO-LUMO energy gaps of glycine interacted with ZnO, MgO, and CaO throughout the NH_2_ functional group are found to be 2.476, 1.976, and 1.651 eV, respectively instead of 6.129 eV. Meanwhile, for the interaction proceeds through the COOH group the HOMO-LUMO energy gaps becomes 2.418, 1.926, and 1.643 eV for the same sequence. The results show that the supposed model molecules exhibit bandgaps ranging from 1.643 to 2.476 eV. Because of the smaller values of ΔE, electrons are more likely to be stimulated from the ground state to the excited one.

**Fig. 4:**
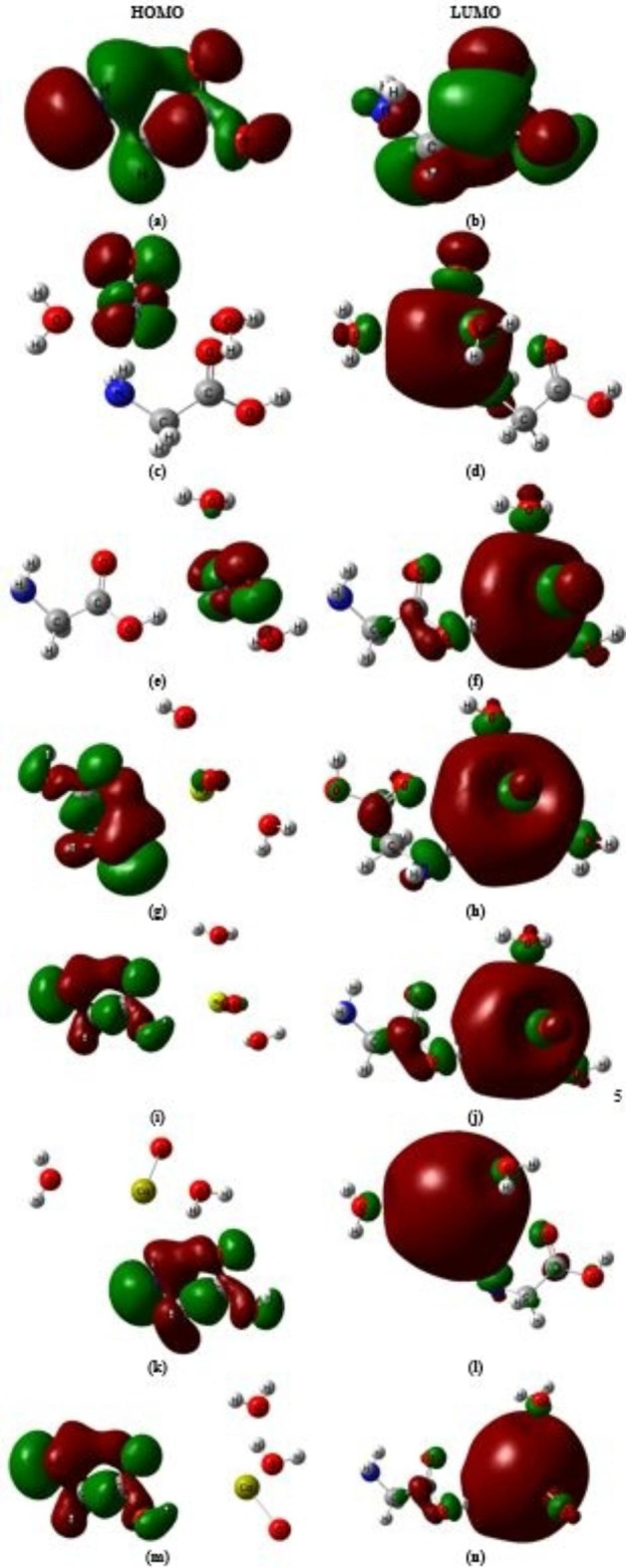
B3LYP/6-31G(d, p) calculated HOMO and LUMO orbitals of (**a**) and (**b**) glycine, (**c**) and (**d**) Glycine (NH_2_)-ZnO-H_2_O, (**e**) and (**f**) Glycine (COOH)-ZnO-H_2_O, (**g**) and (**h**) Glycine (NH_2_)-MgO-H_2_O, (**i**) and (**j**) Glycine (COOH)-MgO-H_2_O, **k**) and **l**) Glycine (NH_2_)-CaO-H_2_O, and **m**) and **n**) Glycine (COOH)- CaO-H_2_O, respectively.

As a result, the structure representing glycine interacted with hydrated CaO through the COOH group has a lower ΔE value than the other five supposed model molecules. The supposed models’ HOMO charge density is primarily found on the metal oxide in the case of hydrated ZnO, while in the case of MgO and CaO, the HOMO charge density is mostly concentrated on the whole structure as presented in Fig. 4. Meanwhile, the LUMO charge density is primarily found on the is mostly concentrated on the whole structure of the studied models. The figure showed that the charge transfer increased due to the interaction of glycine with ZnO, MgO, and CaO. The high charge transition in the supposed models makes them suitable for sophisticated NLO devices.

### Total density of states (TDOS)

**Fig. 5:**
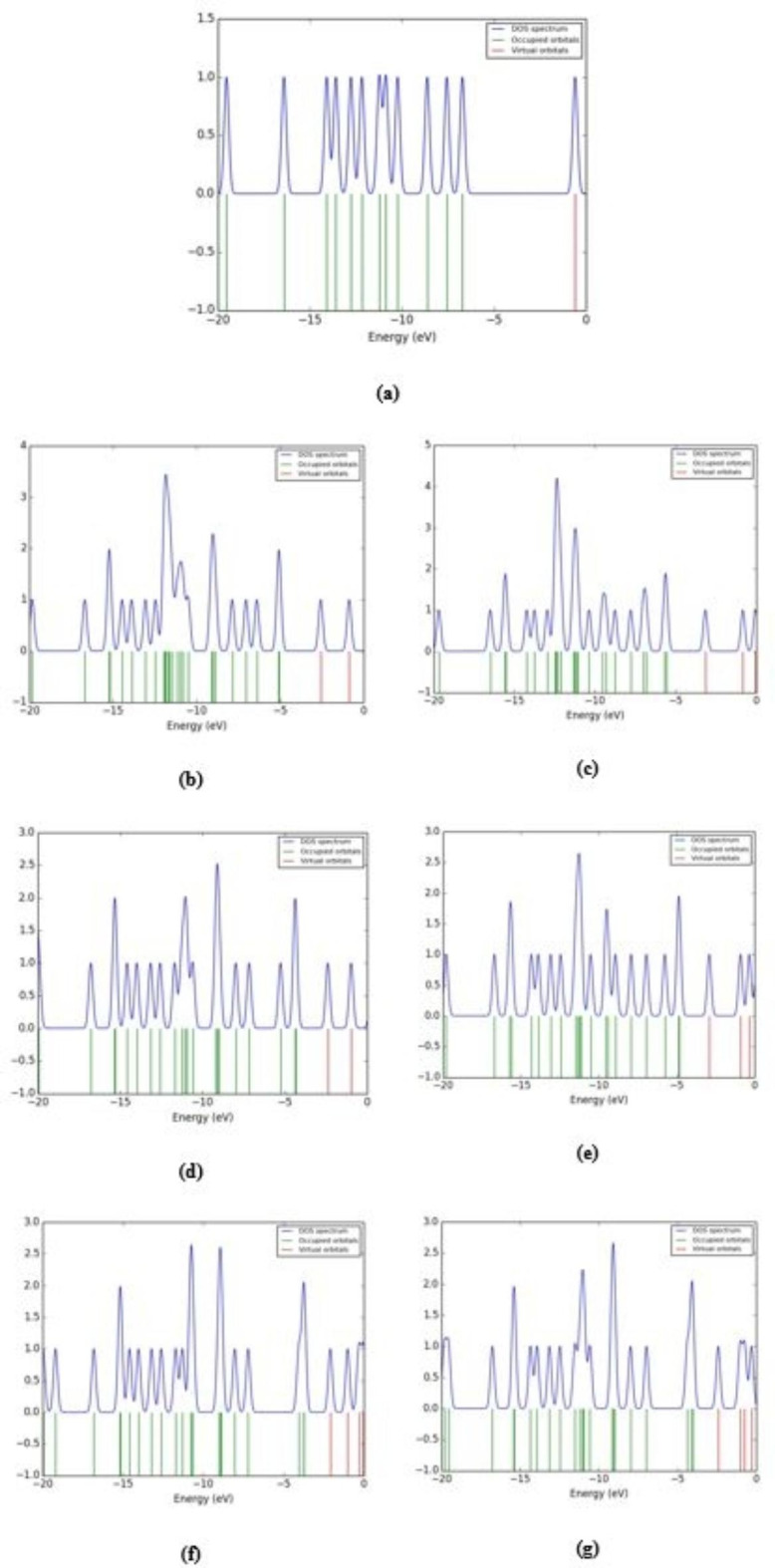
B3LYP/6-31G(d, p) calculated TDOS plots of **a**) glycine and glycine interacted with hydrated ZnO, MgO, and CaO through: **b**), **d**), and **f**) NH_2_ functional group and **c**), **e**), and **g**) COOH functional group.

DOS also known as the number of electron states per unit volume per unit energy refers to the number of distinct states that electrons can occupy at a specific energy level^52^. We investigated the DOS of glycine and glycine interacted with ZnO, MgO, and CaO to better understand the band structure and orbital contributions to valence band maxima (VBM) and conduction band minima (CBM). Moreover, higher electronic band densities help to improve the conductivity of the material by allowing faster electron conduction from outside sources to the reaction sites, which in turn speeds up reaction rates. Furthermore, various electronic band structures can result in varying materials characteristics, and materials types and proportions can be controlled by adjusting the band structure. The B3LYP/6–31G(d, p) calculated TDOS of glycine and glycine interacted with ZnO, MgO, and CaO through the two interaction mechanisms is shown in Fig. 5. As presented in the figure more energy states introduced to the HOMO orbitals of glycine due to its interaction with ZnO, MgO, and CaO confirming the effective transfer of charges.

### Vibrational assignments

**Fig. 6:**
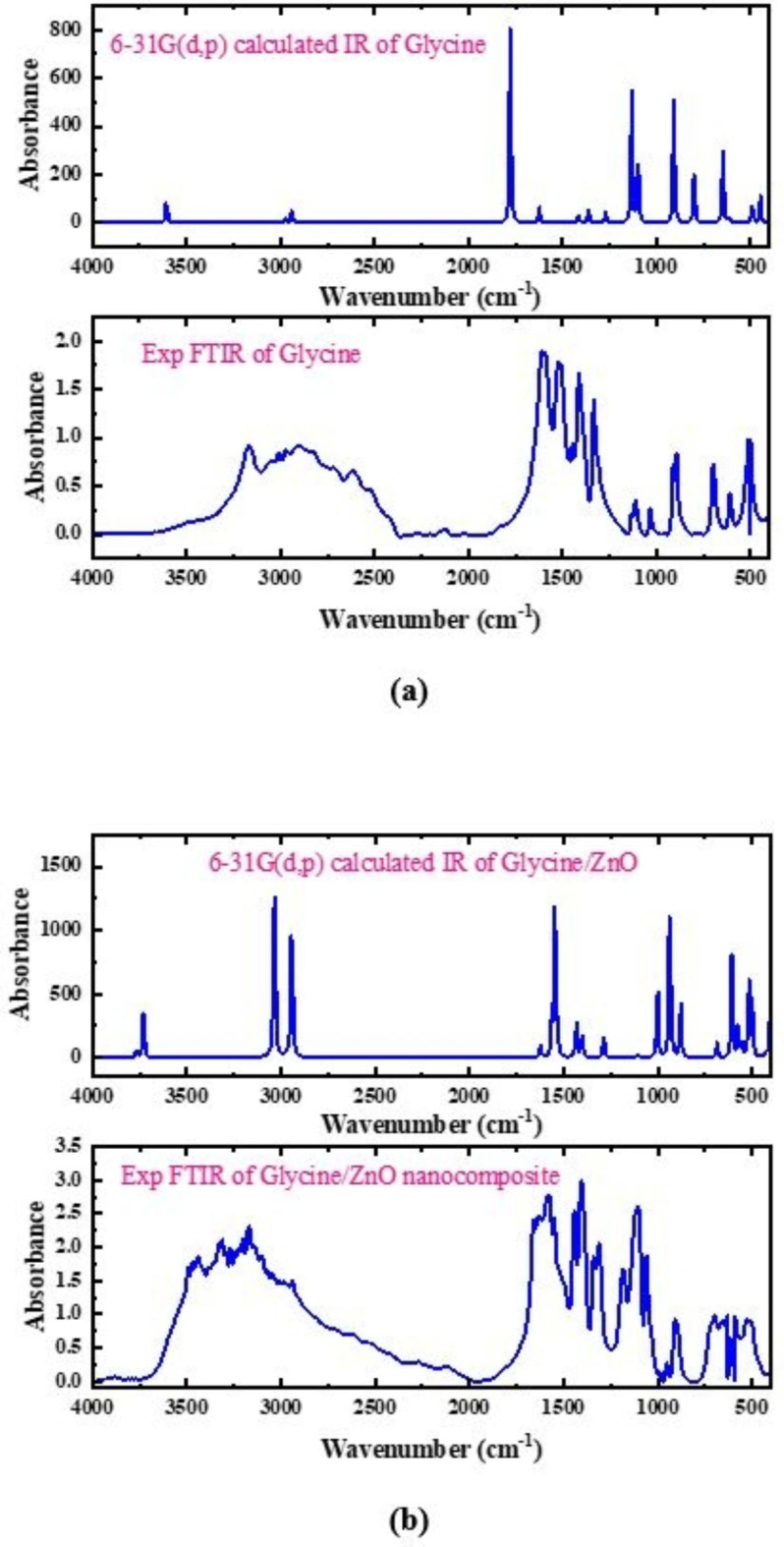
B3LYP/6–31G(d, p) calculated IR and experimental FTIR spectra of (**a**) Glycine and (**b**) Glycine/ZnO nanocomposite.

 Vibrational computational analysis using DFT technique at a suitable basis set is often used for accurate IR band assignment. When an appropriate basis set is used, it is most frequently possible to establish a reasonable correlation between scaled theoretical and experimental wavenumbers. The theoretical wavenumber for a given basis set has been shown to be scaled by a scaled number (0.962), which is indicative of the overestimation of vibrational frequencies in computational simulations. Figure 6 displays the theoretical and experimental infrared spectra of glycine and glycine/ZnO nanocomposites. Table 2 provides an overview of the comparison studies of scaled theoretical and experimental vibrational wavenumbers. The experimental data results and the theoretical results agree rather well. This study presents the assignment of distinct vibrational frequencies to various types of vibrations. There are two types of fundamental modes in vibrational bands: stretching and bending. Bending vibrations include scissoring, whirling, wagging, and rocking. Stretching bands can be symmetric or asymmetric.

The OH stretching vibration of glycine is detected at 3602.952 and 3172 cm^−1^ theoretically and experimentally, respectively. NH_2_ asymmetrical stretching was detected at 3426.073 and 3008 cm^−1^ for the same sequence. Meanwhile, CH asymmetrical stretching was found at 2971.378 and 2970 cm^−1^, as well as CH symmetrical stretching at 2935.810 and 2909 cm^−1^. The high intensity bands seen at 1774.560 and 1828 cm^−1^ for the identical sequence are attributed to C = O stretching with C-O-H bending and NH_2_ scissoring. NH_2_ bending vibrations were recorded at 1622.674 and 1609 cm^−1^. Finally, the detected bands ranging from 1358.317 to 488.340 cm^−1^ and from 1331 to 503 cm^−1^ theoretically and experimentally, respectively, are attributed to the NH_2_ + CH_2_ + CO_2_ + C-C-O bending vibrations^53^.

As demonstrated in Fig. 6, for Glycine (COOH)-ZnO-H_2_O model molecule calculated at the 6-31G(d, p), hydrogen bonding interactions dramatically modify the infrared (IR) spectra. These interactions are linked to frequency shifts and increases in IR intensity that correspond to the vibrational modes of the functional groups directly implicated in the hydrogen-bonded bridges.

The OH stretching vibration shifted to 3727.75 and 3694 cm^−1^, as resented in Table 2, for the theoretical and experimental IR, respectively. Meanwhile, the NH_2_ asymmetric and symmetric stretching vibrations shifted to 3431.454 and 3360.266 cm^−1^ and to 3489 and 3312 cm^−1^ for the theoretical and experimental IR, respectively. Additionally, the CH_2_ asymmetric and symmetric stretching vibrations shifted to 2972.58 and 2936.986 cm^−1^ and 3171 and 2941 cm^−1^ for the same sequence.

The intense bands correspond to the NH_2_ and OH bending vibrations were observed theoretically at 1621.932 and 1561.326 cm^−1^ and experimentally at 1661 and 1575 cm^−1^. Vibrations assigned to OH bending with strong C = O stretching were observed theoretically at 1544.972 cm^−1^ and experimentally at 1552 cm^−1^. Meanwhile, the symmetric stretching of C-C and scissoring vibration of CH_2_ observed theoretically at 1433.38 and 1402.596 cm^−1^ and experimentally at 1443 and 1405 cm^−1^. Additionally, the NH_2_ twisting coupled with the CH_2_ twisting vibrations were observed theoretically at 1336.218 and 1137.084 cm^−1^ and experimentally at 1311 and 1185 cm^−1^. Meanwhile, NH_2_ and OH bending vibrations shifted to 1103.414 and 936.988 cm^−1^ for the theoretical IR and to 1106 and 952 for the experimental IR, respectively. NH_2_ + O = C = O wagging vibrations were observed theoretically at 929.292 and 685.906 cm^−1^ and experimentally at 907 and 699 cm^−1^. Finally, the Zn = O asymmetric and symmetric stretching vibrations were observed at 575.276 and 516.594 cm^−1^ theoretically and 615 and 529 cm^−1^ experimentally, respectively.

Table 2 shows a good agreement between the theoretically calculated IR and experimental FTIR spectra of glycine and glycine/ZnO nanocomposites. This indicates that the 6-31G(d, p) basis set is suitable for IR frequency calculations, validating the proposed structures of glycine and glycine/metal oxide model molecules. These data were found to match values reported in the literature^53^.

**Table 2:** The B3LYP/6-31G(d, p) calculated IR spectrum and experimental FTIR spectrum of glycine and glycine/ZnO model.

Glycine			Glycine/ZnO nanocomposite		
Scaled IR	Experimental FTIR	Assignment	Scaled IR	Experimental FTIR	Assignment
3602.952	3172	OH stretching	3727.750	3694	OH stretching
3426.073	3008	NH_2_ asymmetric stretching	3431.454	3489	NH_2_ asymmetric stretching
2971.378	2970	CH asymmetric stretching	3360.266	3312	NH_2_ symmetric stretching
2935.810	2909	CH stretching	2972.580	3171	CH_2_ asymmetric stretching
1774.560	1828	C = O str + C-O-H bending + NH_2_ scissoring	2936.986	2941	CH_2_ symmetric stretching
1622.674	1609	NH_2_ bending	1621.932	1661	NH_2_ bending
1414.14	1412	CH_2_ bending	1561.326	1575	OH bending
1358.317	1331	CH_2_ bending	1544.972	1552	OH bending + C = O stretching
1139.141	1132	NH_2_ + CH_2_ wagging	1433.380	1443	C-C symmetric stretching + CH_2_ scissoring
1095.882	1111	C-O-H bending + NH_2_	1402.596	1405	C-C symmetric + CH_2_ scissoring
905.545	910	NH_2_ + CH_2_ wagging	1336.218	1311	NH_2_ + CH_2_ twisting
796.918	889	NH_2_ wagging + CO_2_ bending	1137.084	1185	NH_2_ + CH_2_ wagging
644.071	697	NH_2_ + CH_2_ twisting	1103.414	1106	NH_2_ bending
488.340	503	NH_2_ + CH_2_ torsion	936.988	952	OH bending
			929.292	907	NH_2_ + O = C = O wagging
			685.906	699	NH_2_ + O = C = O twisting
			575.276	615	Zn = O asymmetric stretching
			516.594	529	Zn = O symmetric stretching

### UV–Visible absorption studies

**Fig. 7:**
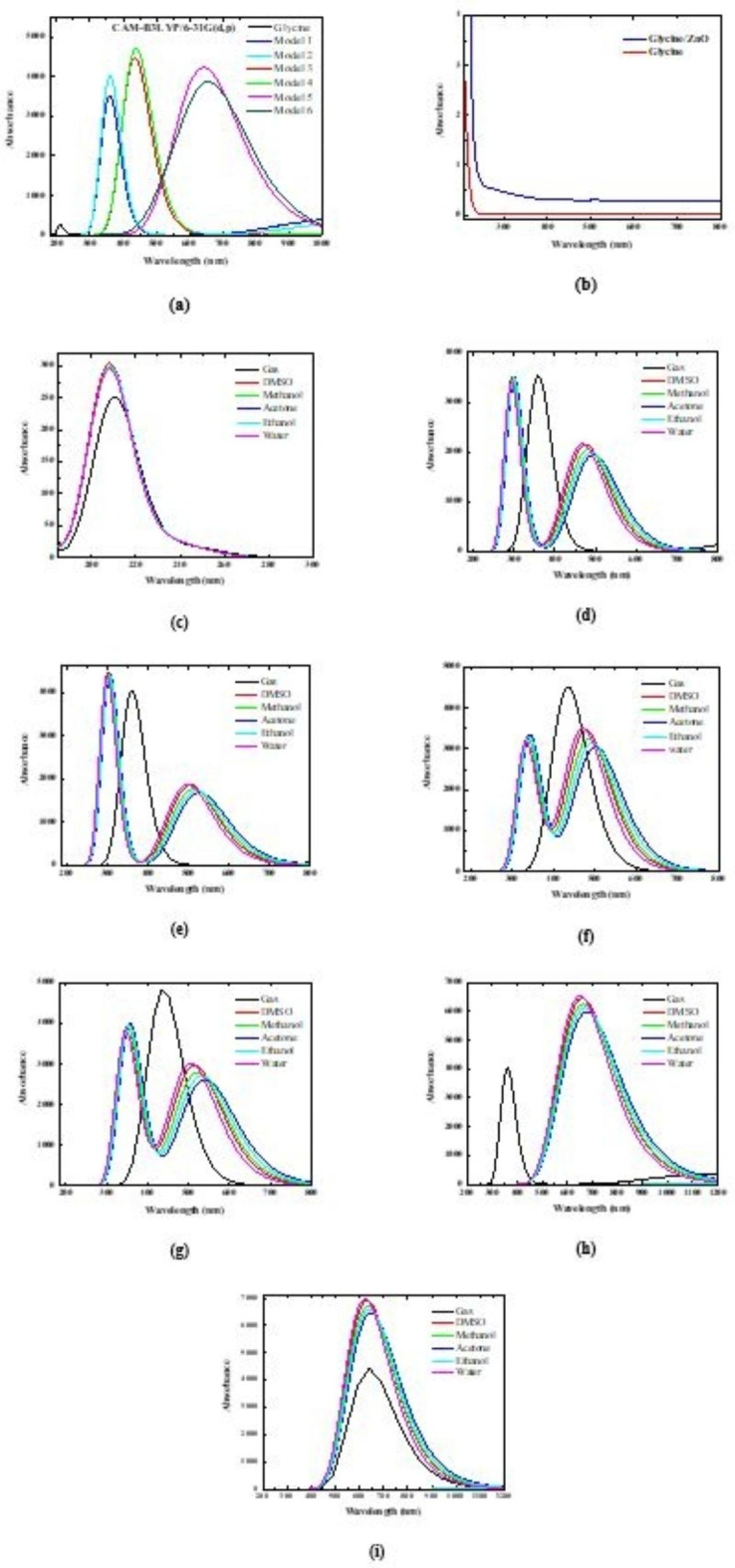
**(a)** TD-DFT/CAM-B3LYP/6–31G(d, p) calculated UV – vis. absorption spectra of Glycine, Glycine (NH_2_)-ZnO-H_2_O (Model 1), Glycine (COOH)-ZnO-H_2_O (Model 2), Glycine (NH_2_)-MgO-H_2_O (Model 3), Glycine (COOH)-MgO-H_2_O (Model 4), Glycine (NH_2_)-CaO-H_2_O (Model 5), and Glycine (COOH)- CaO-H_2_O (Model 6), **(b)** experimental UV – vis. absorption spectra of glycine and glycine/ZnO nanocomposite, and the effect of different solvents on **(c)** glycine, **(d)** Model 1, **(e)** Model 2, **(f)** Model 3, **(g)** Model 4, **(h)** Model 5, and** (i)** Model 6.

Utilizing the CAM-B3LYP/6–31G(d, p) theoretical level and the TD-DFT approach, the study set out to determine the maximum wavelengths, oscillator power, and configurations of glycine nanocomposite structures. Its computational calculations were carried out in the gas phase. Using the optimized structure, the absorption wavelength which indicates electron excitation from HOMO to LUMO was computed. The investigated molecule’s theoretical and experimental UV–vis spectra are displayed in Fig. 7-a and b. Additionally, Fig. 7-c to Fig. 7-h shows the effect of different solvents on the UV-Vis absorption spectra of glycine, Glycine (NH_2_)-ZnO-H_2_O, Glycine (COOH)-ZnO-H_2_O, Glycine (NH_2_)-MgO-H_2_O, Glycine (COOH)-MgO-H_2_O, Glycine (NH_2_)-CaO-H_2_O, and Glycine (COOH)-CaO-H_2_O, respectively, calculated at the DFT/CAM-B3LYP/6-31G(d, p) level of theory. The intensity of the absorption peaks was affected strongly due to the presence of in the presence of the five representative solvents: water, methanol, ethanol, DMSO, and acetone. The UV–vis. absorption spectrum of glycine is shifted to the lower wavelength region due to the addition of solvent and exhibit only one band of maximum absorption at 208 nm for all solvents. However, two absorption bands appeared for the different model molecules representing glycine/ metal oxide, one belongs to the glycine and the higher wavelength band belongs to the solvent. The glycine’ band of maximum absorption strongly shifted to the lower wavelength region with increasing solvent polarity.

Additionally, it can be seen clearly in Fig. 7-c that water, methanol, DMSO and acetone all make the UV-Vis absorption value improved for glycine. Among them, the DMSO is the most obvious, and the UV-Vis absorption peak has a more obvious red shift. While in water solvent, the absorption peak is significantly reduced. At the same, it can be seen clearly in Fig. 7-d that DMSO and acetone all make the absorption peak increase to a certain extent and the presence of water solvent reduced the peak intensity for Glycine (NH_2_)-ZnO-H_2_O and Glycine (COOH)-ZnO-H_2_O model molecules. While for Glycine (NH_2_)-MgO-H_2_O and Glycine (COOH)-MgO-H_2_O model molecules the intensity of the UV-Vis absorption peak in the gas phase is higher than that in the studied solvents. Moreover, intensity of the UV-Vis absorption peak in the acetone solvent is the highest and in water solvent it significantly reduced. Finally, for Glycine (NH_2_)-CaO-H_2_O and Glycine (COOH)-CaO-H_2_O model molecules. However, for Glycine (NH_2_)-CaO-H_2_O and Glycine (COOH)-CaO-H_2_O model molecules, the absorption peak was shifted strongly to the higher wavelength region and the intensity increased due to the solvent addition. The intensity increased with increasing polarity.

Table 3 shows the excitation energy, absorption wavelengths (in nm), oscillator strength (ƒ), and the electronic configuration at the TD-DFT/CAM-B3LYP/6-31G(d, p) level of theory for glycine interacted with ZnO, MgO, and CaO, including. As a well-known quantum mechanical technique, TD-DFT is frequently used for dealing with the electronic excitation of massive systems, including both organic and inorganic systems^45^. Because of its cheap computational cost, TD-DFT is very practical and efficient. The use of the B3LYP hybrid functional allows for the prediction of excitation energies for singlet excitation with a high degree of accuracy, provided that the exchange–correlation functional contains the appropriate functional. The ground and singlet excited state geometries can be represented at the chosen theoretical level. Three lowest singlet excitations were taken into consideration in order to excite the optimized S_0_ geometry of the proposed structures.

As presented in Table 3, the entire model molecules’ excitation energy decreased due to the interaction of glycine with ZnO, MgO, and CaO, in the gaseous phase, indicating that electron excitation was simple. Electronic excitation energy decreases to 5.091 eV from 1.9174 eV. When molecular transitions move to larger percentages, it is easier to excite electrons from the highly occupied molecular orbitals to the lowest unoccupied molecular orbitals (HOMO-LUMO) energy state.

As presented in the table, the initial singlet excited state absorption wavelength for glycine is 243.52 nm, and the oscillator strength is 0.0004. Meanwhile, the second and third excited states absorption wavelengths for glycine are 211.05 and 162.43 nm with the oscillator strength of 0.0063 and 0.0496, respectively. The high intensity absorption band of glycine has been observed at 359.5, 360.63, 435.32, 438.38, 639.54 and 646.64 nm with oscillator strengths of 0.0877, 0.1001, 0.112, 0.1199, 0.1096, and 0.1088 for Glycine (NH_2_)-ZnO-H_2_O, Glycine (COOH)-ZnO-H_2_O, Glycine (NH_2_)-MgO-H_2_O, Glycine (COOH)-MgO-H_2_O, Glycine (NH_2_)-CaO-H_2_O, and Glycine (COOH)-CaO-H_2_O, respectively.

For verification of the models, the UV-vis. absorbance spectra of glycine and glycine/ZnO nanocomposite was measured experimentally. A red shift has been associated with an increase in polarity following an increase in absorption intensity was observed in Fig. 7 for both the experimental and theoretical UV-vis. absorbance spectra. This means that the theoretical and experimental UV-vis. absorbance spectra are in good agreement. Table 3 presents the red shift in the absorption edge for the theoretically UV-vis. absorbance spectra. As presented in the table all bands are attributed to (π-π*) and (n-π*) transitions. This indicates that the excited state stabilizes more as the polarity increases.

**Table 3:** TD-DFT/CAM-B3LYP/6–31G(d, p) computed excitation energies (in eV), transition type, maximum wavelength (in nm), oscillator strengths (ƒ), and electronic transition configurations for the optical transitions of the absorption bands in the UV–vis. Regions.

Structure	Transition	Excitation energies (eV)	Type of transition	λ_max_ (nm)	Oscillator strengths (ƒ)	Configuration composition corresponding transition orbital
Glycine	S0—*>*S1	5.091	π-π*	243.52	0.0004	19 A->21 A (0.69); 20 A->21 A (-0.128)
	S0—*>*S2	5.875	π-π*	211.05	0.0063	20 A->21 A (0.68895); 19 A->21 A (0.12725)
	S0—*>*S3	7.633	π-π*	162.43	0.0496	18 A->21 A (0.63281); 17 A->21 A (-0.18826); 19 A->22 A (-0.17312)
Glycine (NH_2_)-ZnO-H_2_O	S0—*>*S3	3.449	π-π*	359.50	0.0877	47 A->50 A (0.64666); 48 A->53 A (-0.13325); 49 A->52 A (0.11664); 48 A->52 A (-0.1046)
Glycine (COOH)-ZnO-H_2_O	S0—*>*S3	3.438	π-π*	360.63	0.1001	47 A->50 A (0.62866); 48 A->52 A (0.21102); 49 A->53 A (0.11921)
Glycine (NH_2_)-MgO-H_2_O	S0—*>*S3	2.848	n → π∗	435.32	0.112	38 A->41 A (0.63305); 40 A->43 A (0.18189); 39 A->44 A (0.1449); 40 A->41 A (-0.1296); 40 A->44 A (0.10106)
Glycine (COOH)-MgO-H_2_O	S0—*>*S3	2.828	n → π∗	438.38	0.1199	38 A->41 A (0.61608); 39 A->43 A (0.179); 40 A->43 A (0.13263); 39 A->41 A (-0.12346); 40 A->44 A (-0.1155); 40 A->41 A (-0.11519); 39 A->42 A (0.10647)
Glycine (NH_2_)- CaO-H_2_O	S0—*>*S3	1.939	n → π∗	639.54	0.1096	4 A->45 A (0.57998); 42 A->45 A (0.2804); 42 A->47 A (-0.15952); 44 A->49 A (0.13866); 43 A->47 A (-0.12577); 43 A->48 A (0.1154); 42 A->48 A (-0.11497)
Glycine (COOH)- CaO-H_2_O	S0—*>*S3	1.917	n → π∗	646.64	0.1088	44 A->45 A (0.59611); 42 A->45 A (0.24204); 42 A->46 A (0.2269); 43 A->48 A (0.14746); 44 A->49 A (0.13216)

#### Structure activity relationship (SAR)

The biological activity of glycine and glycine interacted with ZnO, MgO, and CaO can be connected to the projected ground state energy and chemical reactivity properties. The energy gap computed at B3LYP/6–31G(d, p) can theoretically explain the chemical reactivity of glycine and its nanocomposites. Table 4 lists the global reactivity descriptors of glycine and glycine interacted with ZnO, MgO, and CaO. The Koopmans hypothesis was used to investigate the global reactivity descriptors. Ionization energy (IE), electron affinity (EA), chemical potential (µ), chemical hardness (**η**), chemical softness (S), electrophilicity index (ω), electronegativity (χ), neuclophilicity index(N), and electronic charges (**∆**N_max_) were among the parameters that were calculated using frontier molecular orbital energies. The study found that the model molecule representing Glycine (COOH)-CaO-H_2_O has the smallest band energy gap (see Table 1), making internal charge transfer easier. In terms of HOMO and LUMO reactivity, this molecule has the most reactive HOMO and LUMO. This means that the maximum charge transfer is occurring in this model.

Chemical substances’ capacities to accept and donate electrons are determined by measuring their IE and EA^54^. These metrics are used to determine the electrophilic strength; higher values of hardness (η) and chemical potential (µ) suggest increased kinetic stability. These variables also show an inverse association with global softness (σ) and are directly related to ΔE values. As a result, molecules with a smaller ΔE are more polarization-sensitive, reactive, and they compete more finely to produce the best NLO response^55^. The results for the calculated global reactivity properties of the investigated models are displayed in Table 4.

From Table 4, the models representing glycine interacted with CaO through both the NH_2_ and COOH functional groups exhibits the lowest ionization energy (IE) value among the proposed structures (3.734 and 4.057 eV, respectively). In contrast, glycine exhibits the highest IE value of 6.712 eV. The ionization potential values follow the order Glycine (NH_2_)-CaO-H_2_O < Glycine (COOH)- CaO-H_2_O < Glycine (NH_2_)-MgO-H_2_O < Glycine (COOH)-MgO-H_2_O < Glycine (NH_2_)-ZnO-H_2_O < Glycine (COOH)-ZnO-H_2_O < glycine.

Additionally, the model molecule representing Glycine (COOH)-ZnO-H_2_O possess the highest electron affinity (EA) value of 3.108 eV, while the structure representing Glycine (NH_2_)-CaO-H_2_O has the lowest value of 2.083 eV. It is notable that for the studied model molecules, the IE values are typically higher than the EA values. Higher IE values are associated with greater chemical stability and inertness, according to the literature^56^.

Chemical potential (µ) is a measure of molecule stability that correlates with electronegativity^57^. A negative µ value indicates that the molecule quickly absorbs electrons^58^. Additionally, the proposed structures have negative chemical potential values, indicating their stability. Chemical potential (µ) follows the order: Glycine (COOH)-ZnO-H_2_O < Glycine (COOH)-MgO-H_2_O < Glycine (NH_2_)-ZnO-H_2_O < glycine < Glycine (NH_2_)-MgO-H_2_O < Glycine (NH_2_)-CaO-H_2_O < Glycine (COOH)- CaO-H_2_O.

The order of chemical hardness (η) is Glycine (COOH)-CaO-H_2_O < Glycine (NH_2_)- CaO-H_2_O < Glycine (COOH)-MgO-H_2_O < Glycine (NH_2_)-MgO-H_2_O < Glycine (COOH)-ZnO-H_2_O < Glycine (NH_2_)-ZnO-H_2_O < glycine correlates with the decreasing energy gap. This indicates that softer molecules with lower ΔE values exhibit lower stability and higher reactivity and vice versa.

Chemical softness (S) is also related to chemical potential (µ) and plays a role in understanding stability and reactivity. The decreasing order of softness is given as Glycine < Glycine (NH_2_)-ZnO-H_2_O = Glycine (COOH)-ZnO-H_2_O < Glycine (NH_2_)-MgO-H_2_O < Glycine (COOH)-MgO-H_2_O < Glycine (NH_2_)-CaO-H_2_O < Glycine (COOH)-CaO-H_2_O. The ascending order contrasts with the growing energy gap order, with Glycine (NH_2_)-ZnO-H_2_O (2.476 eV) having the lowest reactivity and Glycine (COOH)-CaO-H_2_O (1.643 eV) having the highest reactivity and softness among the studied structures.

As presented in Table 4, the electrophilicity index (ω) (electron-rich molecules) is higher than the nucleophilicity index (**N**) for all the supposed structures. Additionally, the highest electronegativity (χ) belongs to the model representing Glycine (COOH)-ZnO-H_2_O of 4.318 eV. Finally, the additional charge carriers (∆N_max_) are calculated for all the supposed structures and the highest two values belongs to Glycine (COOH)-CaO-H_2_O and Glycine (COOH)-MgO-H_2_O. Overall, the ΔE order and the global reactivity descriptors show a very strong relationship. Low ΔE molecules are admitted to exhibit strong nonlinear optical behavior. The supposed model molecules exhibit extraordinary nonlinear optical (NLO) responses, indicating their potential for substantial optoelectronic and sensors applications.

**Table 4:** B3LYP/6-31G(d, p) computed global reactivity parameters of glycine and glycine/metal oxides including ZnO, MgO, and CaO with two water molecules.

Structure	Phase	IE (eV)	EA (eV)	µ (eV)	η (eV)	S (eV)^−1^	ω (eV)	*N* (eV)^−1^	χ (eV)	∆*N*_max_
Glycine	Gas	6.712	0.583	-3.648	3.065	0.326	2.171	0.461	3.648	1.190
	Water	6.785	0.574	-3.680	3.106	0.322	2.180	0.459	3.680	1.185
	Methanol	6.782	0.575	-3.679	3.104	0.322	2.180	0.459	3.679	1.185
	Ethanol	6.780	0.575	-3.678	3.103	0.322	2.180	0.459	3.678	1.185
	DMSO	6.783	0.574	-3.679	3.105	0.322	2.180	0.459	3.679	1.185
	Acetone	6.779	0.575	-3.677	3.102	0.322	2.180	0.459	3.677	1.185
Glycine (NH_2_)-ZnO-H_2_O	Gas	5.044	2.569	-3.807	1.238	0.808	8.965	0.112	3.807	3.076
	Water	4.53	1.154	-2.842	1.688	0.592	2.392	0.418	2.842	1.684
	Methanol	4.511	1.208	-2.860	1.652	0.606	2.476	0.404	2.860	1.731
	Ethanol	4.501	1.236	-2.869	1.632	0.613	2.520	0.397	2.869	1.757
	DMSO	4.52	1.180	-2.850	1.670	0.599	2.432	0.411	2.85	1.707
	Acetone	4.493	1.261	-2.877	1.616	0.619	2.561	0.390	2.877	1.780
Glycine (COOH)-ZnO-H_2_O	Gas	5.527	3.108	-4.318	1.209	0.827	11.270	0.089	4.318	3.571
	Water	4.646	1.426	-3.036	1.610	0.621	2.863	0.349	3.036	1.886
	Methanol	4.642	1.492	-3.067	1.575	0.635	2.986	0.335	3.067	1.947
	Ethanol	4.641	1.527	-3.084	1.557	0.642	3.054	0.327	3.084	1.981
	DMSO	4.644	1.458	-3.051	1.593	0.628	2.922	0.342	3.051	1.915
	Acetone	4.640	1.557	-3.098	1.542	0.649	3.114	0.321	3.099	2.010
Glycine (NH_2_)-MgO-H_2_O	Gas	4.366	2.391	-3.378	0.988	1.012	5.637	0.177	3.378	3.420
	Water	5.201	1.547	-3.374	1.827	0.547	3.115	0.321	3.374	1.847
	Methanol	5.196	1.606	-3.401	1.795	0.557	3.221	0.310	3.401	1.895
	Ethanol	5.194	1.636	-3.415	1.779	0.562	3.278	0.305	3.415	1.920
	DMSO	5.199	1.576	-3.387	1.811	0.552	3.167	0.316	3.388	1.870
	Acetone	5.191	1.662	-3.426	1.764	0.567	3.327	0.301	3.427	1.942
Glycine (COOH)-MgO-H_2_O	Gas	4.831	2.904	-3.867	0.963	1.038	7.203	0.139	3.867	4.015
	Water	4.646	1.426	-3.036	1.610	0.621	2.863	0.3493	3.036	1.886
	Methanol	5.384	1.92	-3.652	1.732	0.577	3.850	0.260	3.652	2.109
	Ethanol	5.389	1.954	-3.671	1.717	0.582	3.924	0.255	3.672	2.138
	DMSO	5.380	1.886	-3.633	1.747	0.572	3.778	0.265	3.633	2.080
	Acetone	5.393	1.983	-3.688	1.705	0.587	3.989	0.251	3.688	2.163
Glycine (NH_2_)-CaO-H_2_O	Gas	3.734	2.083	-2.909	0.826	1.211	3.492	0.286	2.909	3.523
	Water	3.214	0.848	-2.031	1.183	0.845	1.743	0.574	2.031	1.717
	Methanol	3.21	0.888	-2.049	1.161	0.861	1.808	0.553	2.049	1.765
	Ethanol	3.210	0.910	-2.060	1.150	0.870	1.845	0.542	2.060	1.791
	DMSO	3.212	0.867	-2.039	1.172	0.853	1.774	0.564	2.040	1.739
	Acetone	3.208	0.929	-2.068	1.139	0.878	1.877	0.533	2.069	1.815
Glycine (COOH)- CaO-H_2_O	Gas	4.057	2.414	-3.236	0.822	1.217	4.300	0.233	3.236	3.939
	Water	3.249	0.935	-2.092	1.157	0.864	1.891	0.529	2.092	1.808
	Methanol	3.255	0.986	-2.120	1.134	0.881	1.982	0.505	2.121	1.869
	Ethanol	3.259	1.013	-2.136	1.123	0.890	2.031	0.492	2.136	1.902
	DMSO	3.252	0.959	-2.106	1.147	0.872	1.933	0.517	2.106	1.836
	Acetone	3.263	1.037	-2.15	1.113	0.898	2.077	0.482	2.150	1.932

Additionally, Table 4 presents the effect of solvent on the biological activity of the studied model molecules. For glycine, IE, η, ω (constant), χ increased, while µ, S, N, ∆N_max_ decreased due to the presence of solvent. Additionally, IE, η, and χ increased with increasing polarity, while ω and S remains constant. Meanwhile, EA does not affect. For Glycine (NH_2_)-ZnO-H_2_O, Glycine (COOH)-ZnO-H_2_O, Glycine (NH_2_)-CaO-H_2_O, and Glycine (COOH)- CaO-H_2_O, IE, S, ω, χ, and ∆N_max_ decreased while, EA, µ, η, and N increased due to solvation. Higher chemical softness and lower chemical toughness values indicate that the molecule is more reactive, hence increasing their biological activity. Meanwhile, for glycine and Glycine (NH_2_)-MgO-H_2_O, Glycine (COOH)-MgO-H_2_O model molecules, IE, ω, η, and χ increased while, EA, µ, S, N, and ∆N_max_ decreased due to solvation. Lower chemical softness and higher chemical toughness values indicate that the molecule is stable, hence decreasing their biological activity.

### Nonlinear optical (NLO) properties

The B3LYP/6–31 g (d, p) model estimated the total static dipole moment µ, average polarizability < α>, anisotropy of polarizability Δα, and average first polarizability < β > utilizing the x, y, and z components from Gaussian 09 W output.

The dipole moment (µ) is a crucial factor in assessing the polarizability of different organic materials, as it measures charge separation. The µ of Glycine, Glycine (NH_2_)-ZnO-H_2_O, Glycine (COOH)-ZnO-H_2_O, Glycine (NH_2_)-MgO-H_2_O, Glycine (COOH)-MgO-H_2_O, Glycine (NH_2_)-CaO-H_2_O, and Glycine (COOH)- CaO-H_2_O is determined to be 0.556, 3.023, 3.708, 3.561, 4.252, 3.754, and 4.104 Debye, respectively.

Model molecules representing Glycine (COOH)-MgO-H_2_O and Glycine (COOH)- CaO-H_2_O have the highest overall value of 4.252 and 4.104 Debye. Meanwhile, Glycine (NH_2_)-ZnO-H_2_O has the lowest dipole moment (3.023 Debye). Table 5 shows that all the glycine/metal oxide model molecules have higher polarity along the Z-axis, with µ_z_ values of 0.988, 3.671, 1.112, 4.233, 1.254, and 3.917 Debye, respectively. Meanwhile, glycine had higher polarity along the Y-axis of 0.503 Debye.

Glycine (COOH)-CaO-H_2_O model molecule exhibited the highest average polarizability (< α > = 14.629 × 10^−24^ esu). In contrast, the Glycine (NH_2_)-ZnO-H_2_O model has the lowest < α > value (7.298 × 10^−24^ esu). This means that the presence of MgO increased electron density and improved electron-withdrawing capacity. It can be seen from the table that, for all the supposed models, there is a greater contribution of α_yy_ and very small contribution of α_zz_, which reflects that the molecules are elongated more towards the Y direction and are more contracted to the Z direction.

Table 5 displays the average polarizability values for Glycine, Glycine (NH_2_)-ZnO-H_2_O, Glycine (COOH)-ZnO-H_2_O, Glycine (NH_2_)-MgO-H_2_O, Glycine (COOH)-MgO-H_2_O, Glycine (NH_2_)-CaO-H_2_O, and Glycine (COOH)- CaO-H_2_O are 3.956 × 10^−24^, 7.298 × 10^−24^, 9.425 × 10^−24^, 8.544 × 10^−24^, 10.628 × 10^−24^, 12.743 × 10^−24^, and 14.629 × 10^−24^ esu, respectively. The HOMO-LUMO energy gap influences molecular polarizability, as reported in the literature^59^. The HOMO-LUMO energy gap is inversely proportional to both linear and nonlinear polarizabilities. Small HOMO-LUMO energy gap materials allow for high linear and nonlinear polarizabilities. The obtained results shows that Glycine (COOH)-CaO-H_2_O model molecule has a smaller energy gap than the other models, which leads to higher polarizability values (linear and nonlinear). When band gap energy decreased, comparing to the pure glycine, the nanocomposites showed dramatically upgraded electron transport properties. The structure representing Glycine (COOH)- CaO-H2O exhibit significantly increase in the linear polarizability (< α˃) (14.629 × 10-24 e.s.u.), the anisotropy of polarizability (Δα) (8.757 × 10-24 e.s.u.), and first hyperpolarizability < β˃ (23.117 × 10-30 e.s.u.), due to less energetic excitations with respect to the glycine structure ((< α˃ =3.956 × 10-24e.s.u., < β˃ =1.049 × 10-24 e.s.u., and < β˃ =8.481 × 10-30 e.s.u.). Accordingly, the model molecule representing Glycine (COOH)-CaO-H_2_O nanocomposite, as designed, showed excellent NLO properties and was found to be useful for building future NLO materials. Meanwhile, the values of the anisotropy of the polarizability are 1.049 × 10^−24^, 0.420 × 10^−24^, 5.064 × 10^−24^, 0.0375 × 10^−24^, 6.025 × 10^−24^, 1.348 × 10^−24^, and 8.757 × 10^−24^ esu for Glycine, Glycine (NH_2_)-ZnO-H_2_O, Glycine (COOH)-ZnO-H_2_O, Glycine (NH_2_)-MgO-H_2_O, Glycine (COOH)-MgO-H_2_O, Glycine (NH_2_)-CaO-H_2_O, and Glycine (COOH)- CaO-H_2_O, respectively.

**Fig. 8:**
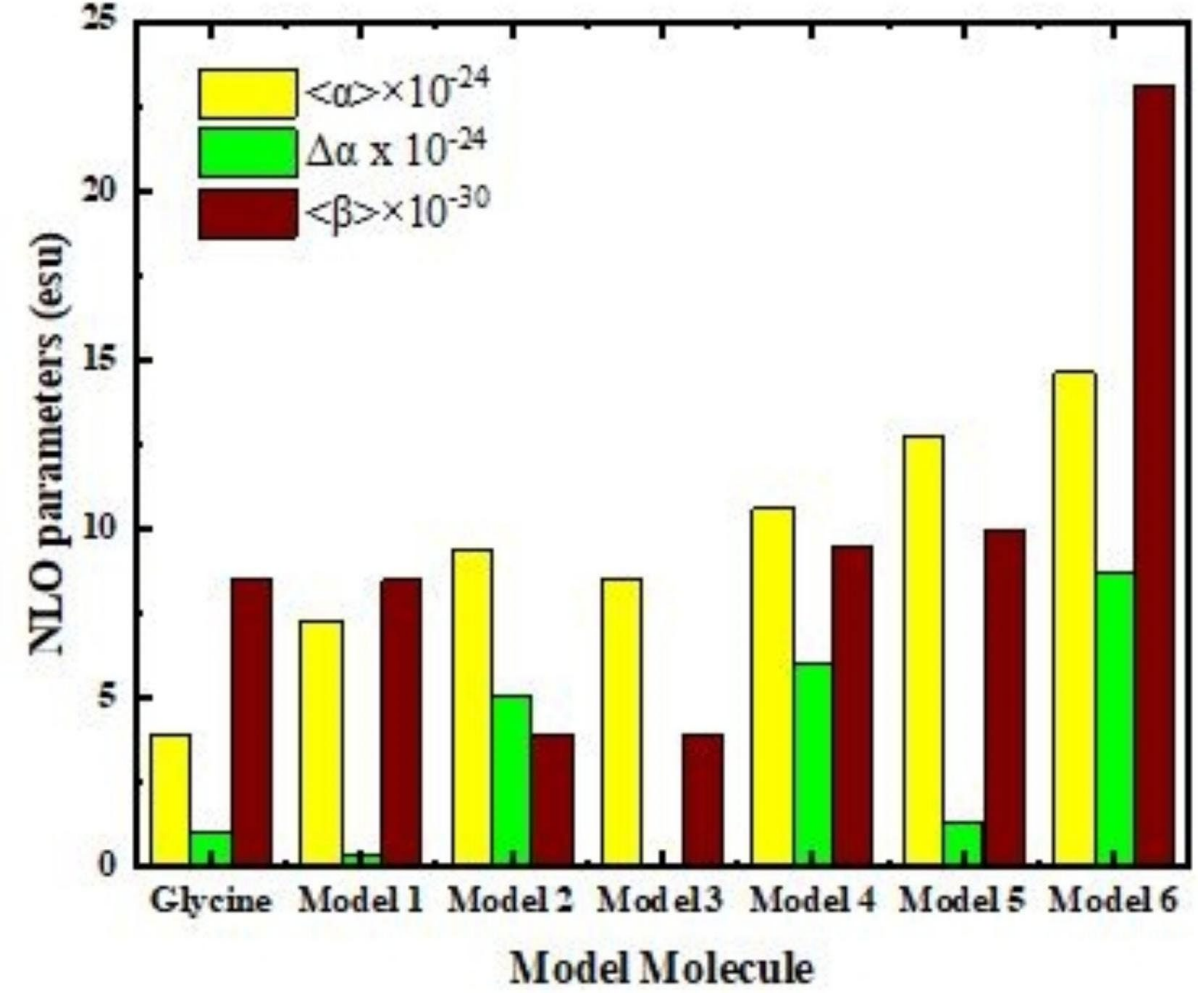
B3LYP/6–31G(d, p) computed NLO parameters of glycine, glycine (NH_2_)-ZnO-H_2_O (Model 1), glycine (COOH)-ZnO-H_2_O (Model 2), glycine (NH_2_)-MgO-H_2_O (Model 3), glycine (COOH)-MgO-H_2_O (Model 4), glycine (NH_2_)-CaO-H_2_O (Model 5), and glycine (COOH)- CaO-H_2_O (Model 6).

The magnitude of the molecule’s first-order hyperpolarizability (< β˃) is a crucial factor in NLO systems. According to the B3LYP/6–31 g (d, p) hypothesis, the model molecule representing Glycine (COOH)-CaO-H_2_O has an initial hyperpolarizability value of 23.117 × 10^−30^ esu. Moreover, the highest two values of β equals 9.937 × 10^−30^ and 23.117 × 10^−30^ esu) which belongs to Glycine (NH_2_)-CaO-H_2_O and Glycine (COOH)-CaO-H_2_O model molecules, respectively. Figure 8 shows the variation of < α˃, (Δα), and < β˃ for glycine, glycine (NH_2_)-ZnO-H_2_O (Model 1), glycine (COOH)-ZnO-H_2_O (Model 2), glycine (NH_2_)-MgO-H_2_O (Model 3), glycine (COOH)-MgO-H_2_O (Model 4), glycine (NH_2_)-CaO-H_2_O (Model 5), and glycine (COOH)- CaO-H_2_O (Model 6).

**Table 5:** Total static dipole moment (µ), mean polarizability ( < α˃), anisotropy of the polarizability (Δα), and the mean first-order hyperpolarizability ( < β˃) for glycine and glycine/metal oxide model molecules at the B3LYP/6–31G(d, p) level.

Property/ Structure	Glycine	Glycine (NH_2_)-ZnO-H_2_O	Glycine (COOH)-ZnO-H_2_O	Glycine (NH_2_)-MgO-H_2_O	Glycine (COOH)-MgO-H_2_O	Glycine (NH_2_)-CaO-H_2_O	Glycine (COOH)- CaO-H_2_O
µ_x_(D)	0.150	-2.816	0.261	-3.348	0.220	-3.518	0.460
µ_y_(D)	0.503	-0.480	-0.450	-0.482	-0.332	-0.380	-1.135
µ_z_(D)	0.182	0.988	3.671	1.112	4.233	1.254	3.917
µ (D)	0.556	3.023	3.708	3.561	4.252	3.754	4.104
α_xx_(a.u.)	36.297	77.390	78.012	92.606	87.731	141.612	115.651
α_xy_(a.u.)	0.337	6.358	-0.441	7.446	1.793	6.493	-0.698
α_yy_(a.u.)	43.377	80.223	112.184	92.859	128.386	132.516	174.740
α_zz_(a.u.)	0.479	-9.740	0.783	-12.327	-0.763	-15.910	6.045
α_yz_(a.u.)	0.352	2.170	-8.560	0.890	-11.083	3.182	-9.376
α_xz_(a.u.)	26.271	99.577	77.735	113.569	92.567	152.404	141.891
<α>×10^−24^(e.s.u.)	3.956	7.298	9.425	8.544	10. 628	12.743	14.629
Δα x 10^−24^(e.s.u.)	1.049	0.420	5.064	0.0375	6.025	1.348	8.757
β_xxx_(a.u.)	123.584	-304.429	-149.619	-709.261	3.847	-2083.206	447.283
β_xxy_(a.u.)	236.093	-15.667	-95.527	17.888	-5.377	112.180	186.315
β_xyy_(a.u.)	555.540	-120.386	10.589	-371.577	-35.963	-862.236	-34.329
β_yyy_(a.u.)	996.798	-166.7903	363.323	-58.195	302.530	250.713	-596.561
β_**xxz**_(a.u.)	19.369	31.233	58.064	21.008	313.281	-82.468	838.578
β_**xyz**_(a.u.)	28.934	23.593	57.328	6.760	59.180	-89.131	10.355
β_yyz_(a.u.)	81.018	20.200	144.160	-7.140	464.252	-186.774	686.538
β_**xzz**_(a.u.)	-7.373	-191.385	13.533	-466.606	15.977	-831.319	28.331
β_**yzz**_(a.u.)	-21.935	13.077	44.482	-90.342	181.989	-364.927	460.814
β_**zzz**_(a.u.)	-0.723	-148.376	340.234	-138.147	776.793	34.321	2306.764
<β>×10^**−30**^**(esu)**	8.481	8.481	3.949	3.900	9.517	9.937	23.117

### Molecular electrostatic potential (MESP)

**Fig. 9:**
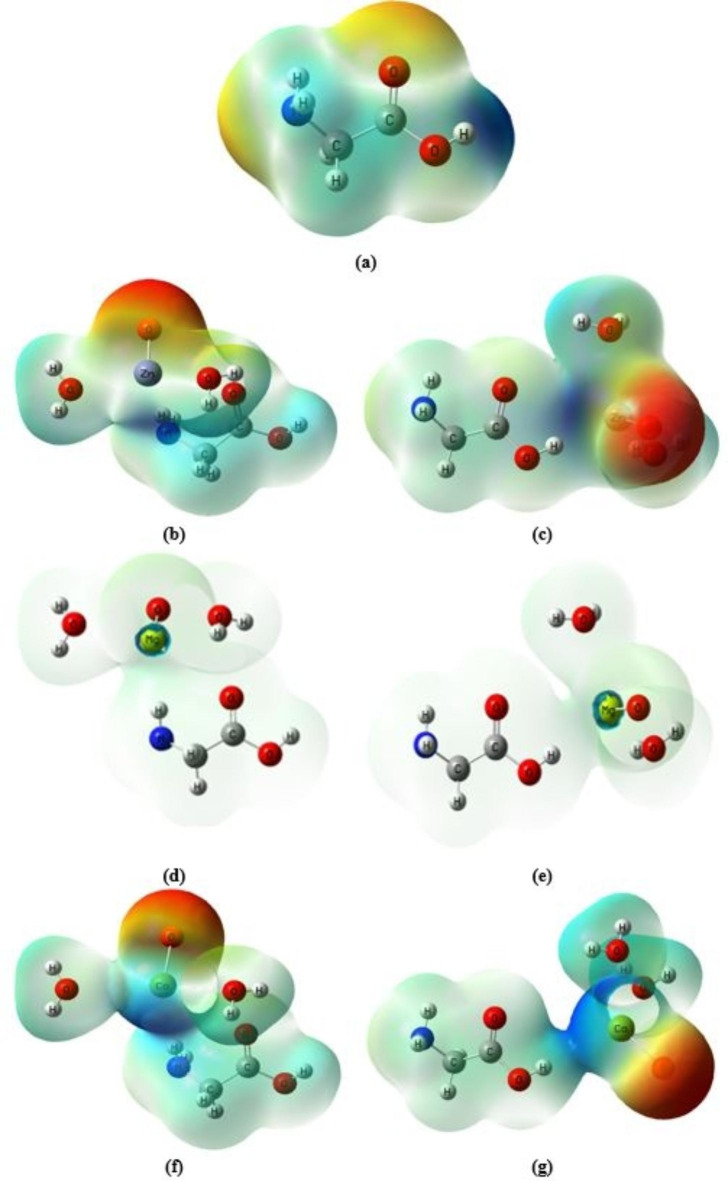
B3LYP/6-31G(d, p) calculated MESP maps as a total density of **a**) glycine and glycine interacted with hydrated ZnO, MgO, and CaO through: **b**), **d**), and **f**) NH_2_ functional group and **c**), **e**), and **g**) COOH functional group.

 The molecular electrostatic potential (MESP) can be used to determine the electrophilic (electron-rich) and nucleophilic (electron-poor) active sites. The MESP shows electron-rich and electron-poor zones in red and blue, and a neutral region in green. A drug’s binding to receptor binding sites is mostly determined by the change in electrostatic potential caused by the molecule, as binding sites typically have opposing electrostatic potentials^60–62^. Figure 9 shows the MESP map of the glycine, glycine/ZnO, glycine/MgO, and glycine/CaO models generated with optimized geometry using Gauss View software at the B3LYP/6–31G(d, p) level. The yellowish-red MESP of the molecule highlights the negative potential region around the oxygen and nitrogen atoms of glycine, as well as the location of attachment for electrophilic assault. Proton H_9_ in the glycine model molecule (attached to COOH group) is the most positively charged, while the remaining protons (H_2_, H_5_, H_6_, and H_10_) in the molecule seem to have a neutral electrostatic potential. However, for glycine interacted with ZnO and CaO throughout the NH_2_ and COOH functional groups, the negative MESP region exists around the oxygen atom of the metal oxide, while the positive potentials exist around the zinc atom. On the other hand, for glycine interacted with MgO, Mg atom of the metal oxide is the most positively charged, while the remaining protons in the molecule have a slightly positive MESP. As presented in the figure the electronic charges are redistributed within the glycine structure due to the interaction with ZnO, MgO, and CaO with increased intensity around the oxygen of the metal oxides. The increased intensity reflects the decrease in the band gap energy hence, increased electronegativity. This confirms the results of the reactivity descriptors especially that the electronegativity increased due to the interaction of glycine with metal oxides.

### Antibacterial activity of the glycine/ZnO nanocomposites

In order to understand the antibacterial properties of glycine/ZnO nanocomposites, the kinetic metabolism curves for each bacterium were examined at various concentrations of nanoparticles. The measurement of optical density (OD) over time has not been frequently utilized in the existing literature to evaluate the effects of nanomaterials (NMs) on bacterial growth^63^.

This study investigates the antibacterial properties and optimal sensitivity of glycine/ZnO nanocomposites against *Staphylococcus aureus* and *Pseudomonas aeruginosa*. The screening outcomes indicated that glycine/ZnO nanocomposites exhibited antibacterial activity against both bacterial strains. Kinetic curves of bacterial metabolism were recorded over a 24-hour period in response to various concentrations of glycine/ZnO nanocomposites. Notable differences were observed between the antibacterial effects of glycine and those of glycine/ZnO nanocomposites. The antibacterial effectiveness was found to increase with higher concentrations of glycine in conjunction with ZnO nanoparticles^64^.

**Fig. 10:**
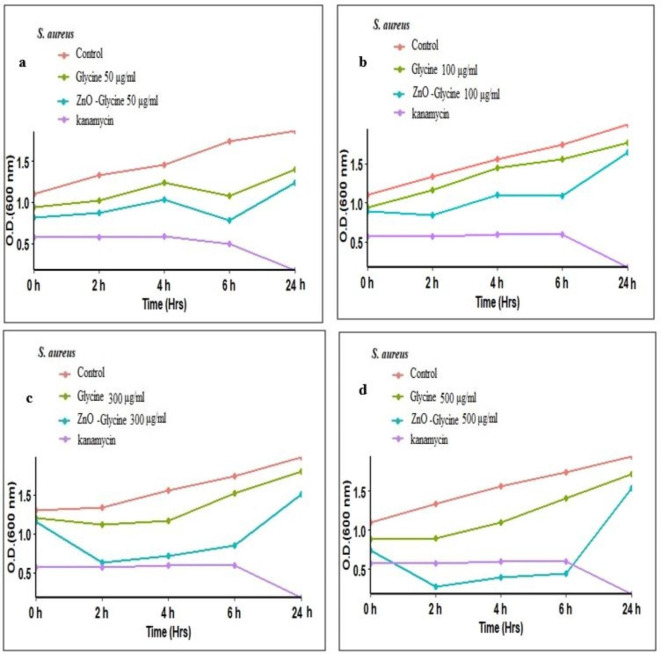
Impact of glycine and glycine/ZnO nanocomposites on the growth of *S. aureus* by measuring optical density (OD) at 600 nm. Bacterial growth curve was generated using various concentrations: **(a)** 50 µg/mL, **(b)** 100 µg/mL, **(c)** 300 µg/mL, and **(d)** 500 µg/mL. Bacteria were treated with a positive control of 100 µg/mL kanamycin and a negative control by incubating in (MHB). The OD measurements represent the mean values from three independent experiments, each performed in triplicate.

**Fig. 11:**
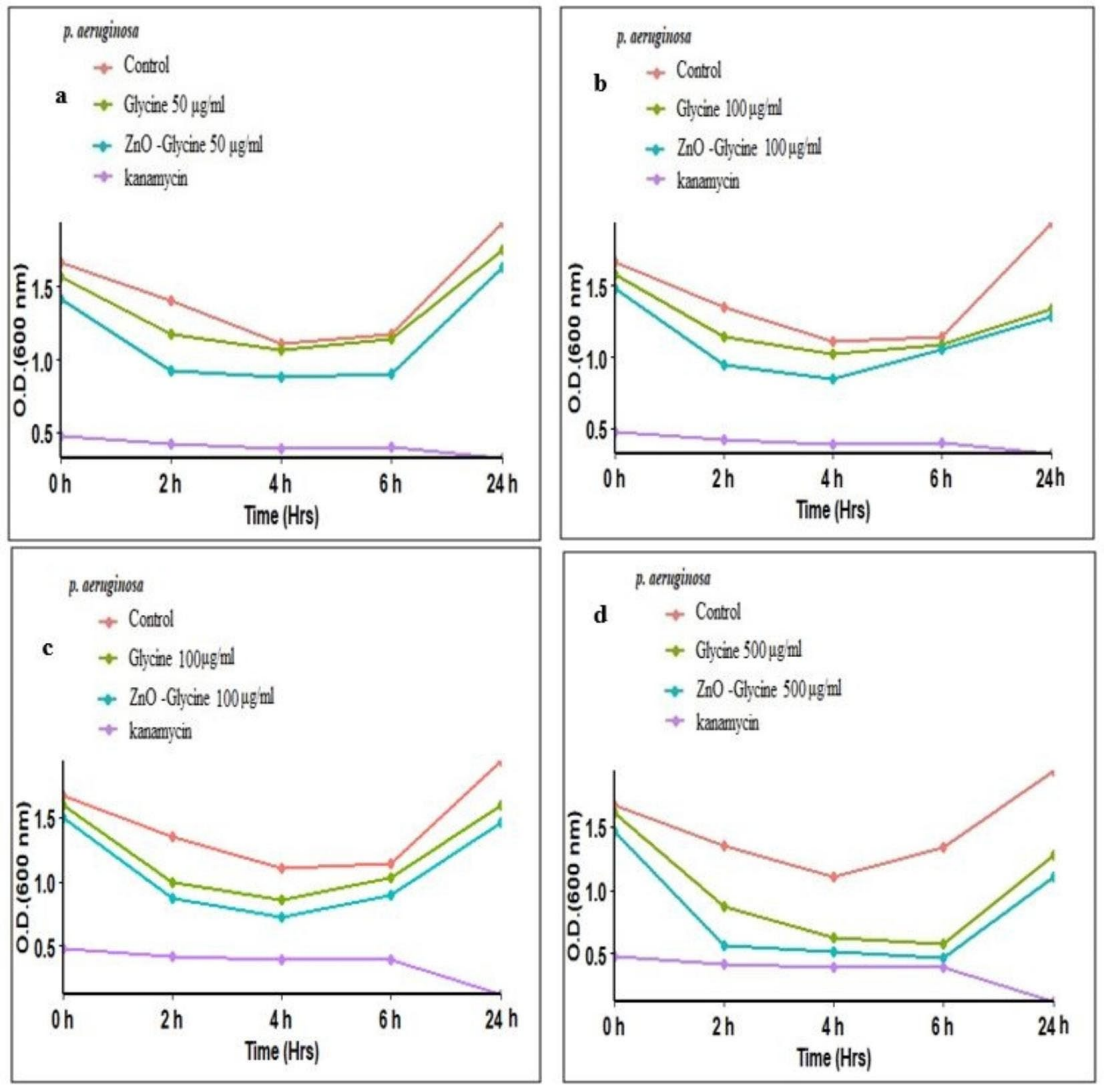
Impact of glycine and glycine/ZnO nanocomposites on the growth of *P. aeruginosa* by measuring optical density (OD) at 600 nm. Bacterial growth curve was generated using various concentrations: **(a)** 50 µg/mL, **(b)** 100 µg/mL, **(c)** 300 µg/mL, and **(d)** 500 µg/mL. Bacteria were treated with a positive control of 100 µg/mL kanamycin and a negative control by incubating in (MHB). The OD measurements represent the mean values from three independent experiments, each performed in triplicate.

 For the control bacteria, there was an observed increase in optical density (OD) over time, indicating active bacterial growth (see Figs. 10 and 11). Exposure to kanamycin effectively prevented any increase in OD, thereby inhibiting bacterial growth at all-time points. While the growth of bacteria in MHB (negative control) increased over time. Glycine/ZnO nanocomposites produced a concentration-dependent decrease in OD for all bacterial strains (see Fig. 10). The most significant growth inhibition was seen in *S. aureus*, with greater effects at higher concentrations of glycine/ZnO nanocomposite and longer exposure durations. *S. aureus* exhibited heightened susceptibility to the toxicity of glycine/ZnO nanocomposites as presented in the figure. At 6 h post-exposure, glycine/ZnO nanocomposites at a concentration of 300 and 500 µg/mL resulted in approximately 50% reduction in growth for both *S. aureus* (Fig. 10-c and d) and *P. aeruginosa* (Fig. 11-c and d) compared to the negative control, respectively. Notably, significant growth inhibition was recorded at concentrations of 300 and 500 µg/mL for both bacterial strains at 2, 4, and 6 h. However, only the 500 µg/mL concentration demonstrated significant inhibition of *S. aureus* growth over the 0–24 h period (Fig. 10-d ). By the end of the experiment (24 h), glycine/ZnO nanocomposites led to a significant decrease in the growth of *S. aureus* at concentrations of both 300 and 500 µg/mL, as indicated by a reduction in optical density. In contrast, significant growth reduction for *P. aeruginosa* was only noted at the 500 µg/mL concentration compared to the control (Fig. 11-d). These results suggest that the growth inhibitory effect of glycine/ZnO nanocomposites can be ranked from most to least sensitive as follows: *Staphylococcus aureus* > *Pseudomonas aeruginosa*. These results suggest that *S. aureus* is more sensitive to the toxicity of glycine/ZnO nanocomposites than *P. aeruginosa*. Our findings indicate that this method facilitates a swift, cost-effective, and high-throughput evaluation of the antibacterial properties of nanocomposites, and we advocate for its broader application. These findings align with the research conducted by^65^, which confirmed the disorganization of Gram-negative membranes through transmission electron microscopy of ultrathin bacterial sections. In essence, prolonged exposure to ZnO nanoparticles results in increasingly detrimental effects on bacterial cells. This mechanism induces pressure within the cell wall, leading to the production of additional lactate dehydrogenase enzymes, which ultimately results in cell membrane lysis, with lethality being contingent upon the duration of exposure. These findings are consistent with earlier studies that demonstrated a time-dependent toxicity of ZnO nanoparticles^66^.

In conclusion, the absorption characteristics demonstrate that adding ZnO, MgO, and CaO as an acceptor to glycine could be suitable to gather additional light on the longer wavelength side. It also helps related solar cell materials’ photo-electrical conversion efficiency. Thus, the field of optoelectronics known as non-linear optics (NLO) has experienced significant growth in recent years, primarily due to the growing demand for high-end technologies like high definition (HD) displays, high-speed optical fibers for transmission as well as combined lasers for medical applications. As a result, current fundamental and applied research is centered on materials with improved NLO features. Additionally, NLO materials with an organic molecular framework are preferred over those inorganic. This can be explained by the inorganic material’s restriction on changing the NLO response. On the other hand, organic material offers structural flexibility and the capacity to adjust in order to optimize NLO response.

## Conclusion

Optical biosensors offer faster analytical speed and real-time analysis, making illness screening easier and early pathogen detection more likely. Researchers must develop reliable biosensors for everyday applications. Accordingly, in order to develop optical biosensor materials for bacterial detection, the reactivity of glycine interacted with metal oxides (ZnO, MgO, and CaO) was investigated using DFT/B3LYP/6-31G(d, p) level of theory. The structure and electronic characteristics of glycine as well as the ways in which it interacted with ZnO, MgO, and CaO molecules were ascertained by a variety of quantum chemical calculations. The results show that the TDM of glycine increased and the bandgap energy decreased due to the interaction with the studied metal oxides. According to the TDM and FMO data, the model molecules representing glycine interacted with CaO possess the lowest band gap energies. The smaller band gap will aid in the efficient transfer of charges. Among the five solvents of water, methanol, ethanol, DMSO and acetone, the water and DMSO solvents enhances the UV-Vis absorption effect. DOS pictographs give further support to the smooth movement of electronic cloud. The supposed structures were investigated using FTIR and UV-vis. spectrophotometers. These spectrum investigations provided critical data to support the structural integrity of the supposed models. Certain bands connected to the NH_2_ and COOH functional groups were validated by IR spectra. The UV-Vis spectral investigations of glycine and glycine interacted with ZnO, MgO, and CaO molecules were conducted theoretically and experimentally. To examine the electronic transitions of the studied model molecules, TD-DFT calculations were carried out on electronic absorption spectra in the gas phase. The results of the experiments were compared with the calculations of the electronic properties, such as the electron transitions and UV-vis. absorption spectra. The UV–vis. absorption spectra of glycine model molecule undergo a red shift due to the interaction with ZnO, MgO, and CaO with increased absorption intensity. The theoretical calculated IR and UV-vis. Absorption spectra are in good agreement with the experimental results. Furthermore, the band gap was also shown to be connected with the global reactivity parameters; model molecules representing glycine interacted with CaO had the lowest global hardness, and the highest softness. According to the NLO study, the newly supposed model molecules have significant advantages over the glycine molecule in technological applications. Additionally, the results show that the models representing glycine interacted with CaO have a higher NLO parameters than the other studied model molecules. The MESP maps show that positive potential sites are located around hydrogen atoms, and negative potential sites are located around oxygen and nitrogen atoms. In summary, the findings of the study indicated that the growth inhibition was dose-dependent, correlating with the oxidative stress effects of glycine/ZnO nanocomposites on *S. aureus* and *P. aeruginosa*. Additionally, it was observed that nanocomposites at higher concentrations exhibited greater antibacterial activity than those at lower concentrations. Gathering the above findings, it is clear that, the investigated electronic structure, global reactivity descriptors and nonlinear optical properties are dedicating Glycine interacted with ZnO, MgO and CaO for effective bacterial detection. More precisely glycine/ZnO nanocomposites hold promise as potential antibacterial agents.

## Data Availability

The data will be available upon request. Contact Medhat A. Ibrahim, Email: ma.khalek@nrc.sci.eg.
